# Widespread loss of mammalian lineage and dietary diversity in the early Oligocene of Afro-Arabia

**DOI:** 10.1038/s42003-021-02707-9

**Published:** 2021-10-07

**Authors:** Dorien de Vries, Steven Heritage, Matthew R. Borths, Hesham M. Sallam, Erik R. Seiffert

**Affiliations:** 1 Ecosystems and Environment Research Centre, School of Science, Engineering and Environment, University of Salford, Manchester, UK; 2 Interdepartmental Doctoral Program in Anthropological Sciences, Stony Brook University, Stony Brook, NY 11794 USA; 3 Duke Lemur Center Museum of Natural History, Durham, NC 27705 USA; 4 Mansoura University Vertebrate Paleontology, Department of Geology, Faculty of Science, Mansoura, Egypt; 5 Institute of Global Health and Human Ecology (I-GHHE), School of Sciences and Engineering, American University in Cairo, New Cairo, Egypt; 6 Department of Integrative Anatomical Sciences, Keck School of Medicine of USC, University of Southern California, Los Angeles, CA USA; 7 Department of Mammalogy, Natural History Museum of Los Angeles County, Los Angeles, CA 90007 USA

**Keywords:** Palaeontology, Phylogenetics

## Abstract

Diverse lines of geological and geochemical evidence indicate that the Eocene-Oligocene transition (EOT) marked the onset of a global cooling phase, rapid growth of the Antarctic ice sheet, and a worldwide drop in sea level. Paleontologists have established that shifts in mammalian community structure in Europe and Asia were broadly coincident with these events, but the potential impact of early Oligocene climate change on the mammalian communities of Afro-Arabia has long been unclear. Here we employ dated phylogenies of multiple endemic Afro-Arabian mammal clades (anomaluroid and hystricognath rodents, anthropoid and strepsirrhine primates, and carnivorous hyaenodonts) to investigate lineage diversification and loss since the early Eocene. These analyses provide evidence for widespread mammalian extinction in the early Oligocene of Afro-Arabia, with almost two-thirds of peak late Eocene diversity lost in these clades by ~30 Ma. Using homology-free dental topographic metrics, we further demonstrate that the loss of Afro-Arabian rodent and primate lineages was associated with a major reduction in molar occlusal topographic disparity, suggesting a correlated loss of dietary diversity. These results raise new questions about the relative importance of global versus local influences in shaping the evolutionary trajectories of Afro-Arabia’s endemic mammals during the Oligocene.

## Abstract (web summary)

Using time-scaled phylogenies of several endemic clades of small Afro-Arabian mammals, Dorien de Vries et al. infer a steep drop in species numbers around the Eocene-Oligocene transition. This dramatic decline coincides with a global cooling trend as well as the onset of widespread flood basalt volcanism in East Africa, and is found to be correlated with a major loss in dietary diversity.

## Introduction

Deep sea cores consistently preserve evidence for a strong oxygen isotope shift (Oi-1, ~33.5 Ma (million years ago)) just after the Eocene-Oligocene boundary (EOB, 33.9 Ma), the timing of which closely correlates with the expansion of Antarctic glaciation and drops in sea level, calcite compensation depth, and sea surface temperatures^1–4^. These abrupt environmental changes in the marine realm roughly coincide with marked shifts in mammalian community structure on some landmasses, most notably Europe^5,6^ and Asia^7–9^. Overall, however, terrestrial records from the early Oligocene provide geographically mixed evidence for environmental change relative to late Eocene conditions^10,11^. The nature of the Eocene-Oligocene transition (EOT) on the Afro-Arabian landmass has long been enigmatic, but recent paleontological sampling has improved our understanding of this phase^12–17^; this enriched context has inspired proposals of increased geographic provincialism of mammalian communities in the early Oligocene of Africa^13^, and led to the identification of multiple strepsirrhine primate extinctions in northern Africa near the EOB^18^.

The inconsistent signal for early Oligocene cooling across the planet suggests that some areas might have experienced gradual environmental change, if any^10,11^. Detection of a long-term biotic response to such gradual environmental change can be complicated by sampling gaps in the fossil record, but Bayesian tip-dating (BTD) phylogenetic analyses^19^ that incorporate rates of morphological evolution to estimate divergence times among living and extinct taxa^20–27^ can partially overcome this problem by allowing the identification of “ghost” lineages that cross temporal and geographic sampling gaps. Here we employ these methods to study lineage diversity in Afro-Arabian primates and rodents, the most common small mammals in Paleogene fossil deposits of that landmass.

Two clades of rodents dispersed into Afro-Arabia during the Eocene—the now largely arboreal but ancestrally terrestrial Anomaluroidea (the scaly-tails or anomalures), which probably arrived in the early Eocene^27,28^, and terrestrial Hystricognathi, which likely dispersed to Afro-Arabia from Asia in the middle Eocene^25,29^. Arboreal primates mirror this pattern of staggered arrival times, with strepsirrhines likely dispersing from Europe or Asia to Afro-Arabia in the early Eocene, and anthropoids appearing in the middle Eocene following a dispersal from Asia^22,26^. These four clades include a great diversity of living and extinct species that have lived in a wide range of arboreal and terrestrial habitats that could have been impacted by environmental change during the early Oligocene.

## Results and discussion

### Lineage diversity through time

**Fig. 1:**
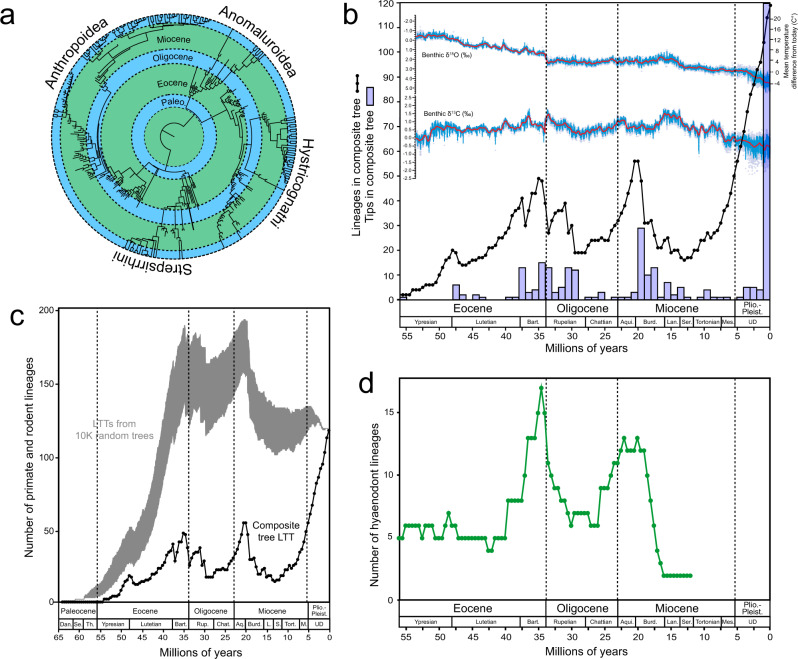
Lineage diversity shifts in Afro-Arabian primates, rodents, and hyaenodonts. **a** Composite time-scaled tree of Afro-Arabian primates and rodents used for calculation of lineage diversity shifts from the Eocene to Recent. See Supplementary Fig. 1 for full tree. **b** Black line is the lineage through time (LTT) plot based on the composite tree in (**a**); light blue histogram shows number of tips in the composite tree in 1 Ma long bins; top, high-resolution astronomically dated benthic carbon (δ^13^C‰) and oxygen (δ^18^O‰) isotope curves from the early Eocene to Recent, from Westerhold et al.^4^. δ^18^O‰ curve provides a proxy for mean temperature difference from the present day. **c** LTT plot for the composite tree compared to 10,000 random trees with the same tip ages as the composite tree, but random topologies and branch lengths. **d** LTT plot for Afro-Arabian Hyaenodonta, a clade of carnivorous placentals that were likely the primary mammalian predators of the primates and rodents in the composite tree. Dan. = Danian, Se. = Selandian, Th. = Thanetian, Bart. = Bartonian, Rup. = Rupelian, Chat. = Chattian, Aq. or Aqui. = Aquitanian, Burd. = Burdigalian, L. or Lan. = Langhian, S. or Ser. = Serravallian, Tort. = Tortonian, M or Mes. = Messian, UD = undivided.

We constructed a near-comprehensive composite time-scaled phylogeny that includes living and extinct members of all four clades, with divergences among extinct taxa based largely on BTD analyses (see “Methods”). The resulting composite tree includes 317 tips, 62% of which (198) are extinct (Fig. 1a and Supplementary Fig. 1). A lineage-through-time (LTT) plot derived from the composite tree (Fig. 1b) reveals three distinct diversity peaks (latest Eocene (35 Ma), earliest Miocene (20–20.5 Ma), and Recent), and two major diversity troughs (mid-Oligocene (28–29.5 Ma) and mid-Miocene (13 Ma)).

The mid-Oligocene and mid-Miocene diversity troughs bottom out near sampling gaps in the Afro-Arabian terrestrial vertebrate fossil record (Fig. 1b), leaving open the possibility that the LTT curves are artificially low during these time periods simply due to a lack of sampling. This explanation can be rejected because the LTT curve shows high lineage diversification during all other periods for which there is little or no paleontological sampling (Fig. 1b). Furthermore, the major losses of lineage diversity that precede the mid-Oligocene and mid-Miocene diversity troughs occur during time periods that are particularly well-sampled^14,16,30^.

We tested whether the magnitude of extinction required by the composite tree could be observed in a randomized sample by generating 10,000 trees that had the same tip ages as those in the composite tree, but random topologies and branch lengths (Fig. 1c). On average, the random trees require a ~17% loss of lineages from peak Eocene diversity to the deepest point in the early Oligocene trough, whereas the composite tree requires a loss of ~61% of lineages across the same interval. The magnitude of the lineage loss observed in the composite tree therefore cannot be explained as an artifact of sampling alone; rather, it is highly dependent on the non-random phylogenetic structure of the composite tree, which reflects a strong lack of phylogenetic continuity (i.e., few ghost lineages) linking the similarly diverse late Eocene and early Miocene primate and rodent communities.

The lineage diversification pattern of Afro-Arabian primates and rodents closely matches that of Afro-Arabian members of Hyaenodonta, a clade of carnivorous mammals that is known to have gone extinct in the late Miocene^23^ (Fig. 1d). Prior to 15 Ma, Afro-Arabian hyaenodonts show the same LTT peaks as are seen in the composite tree of primates and rodents (latest Eocene and early Miocene), with a distinct mid-Oligocene diversity trough (Fig. 1d). Because Hyaenodonta goes extinct in the late Miocene, the veracity of the middle Miocene diversity drop that is also seen in primates and rodents is not open to debate. When hyaenodont lineage numbers are added to those in the composite tree of primates and rodents, the magnitude of total lineage loss from the late Eocene into the mid-Oligocene rises to ~63% (Supplementary Fig. 2). This value closely approximates the estimated worldwide loss of ~67% of all species during the transition from the late Eocene to the early Oligocene^31^.

### Dental topographic disparity through time

The vast majority of the extinct species included in the composite tree of primates and rodents are only known from dental remains, and often only isolated teeth. Fortunately tooth shape reflects diet, and homology-free dental topographic metrics (DTMs) have proven useful for discriminating among primate and rodent species with different dietary preferences^32–34^. More generally, primates and rodents must be able to process food to maintain basic biological functions, so molar dental topography has a strong and unambiguous link to survival. We take disparity in DTM values through time to broadly reflect temporal shifts in dietary breadth. We calculated three DTMs (Dirichlet normal energy (ariaDNE)^35^, orientation patch count rotated (OPCR)^34^, and relief index (RFI)^36^) on lower second molars (M_2_) across a large subset (89%) of the extinct species older than 15 Ma for which M_2_ is known. Lineage counts between 55 and 15 Ma (taken at 0.5 Ma intervals) in the reduced sample on which we calculated DTMs closely track lineage counts in the composite tree (*r*^2^ = 0.97). We calculated ancestral states throughout the tree for the first two principal components (PCs) derived from PC analysis of all three DTMs, and calculated disparity within each major clade at 0.5 Ma intervals (via interpolation) using four different disparity metrics (square root of 2D hull area (SR2DHA), minimum spanning tree length (MST), sum of ranges (SoR), and sum of variances (SoV)). As our estimates of disparity are based on a principal components analysis (PCA) of DTM values, they are only capturing “functional richness” of M_2_ topography within each clade (*sensu* Villeger et al.)^37^.

**Fig. 2:**
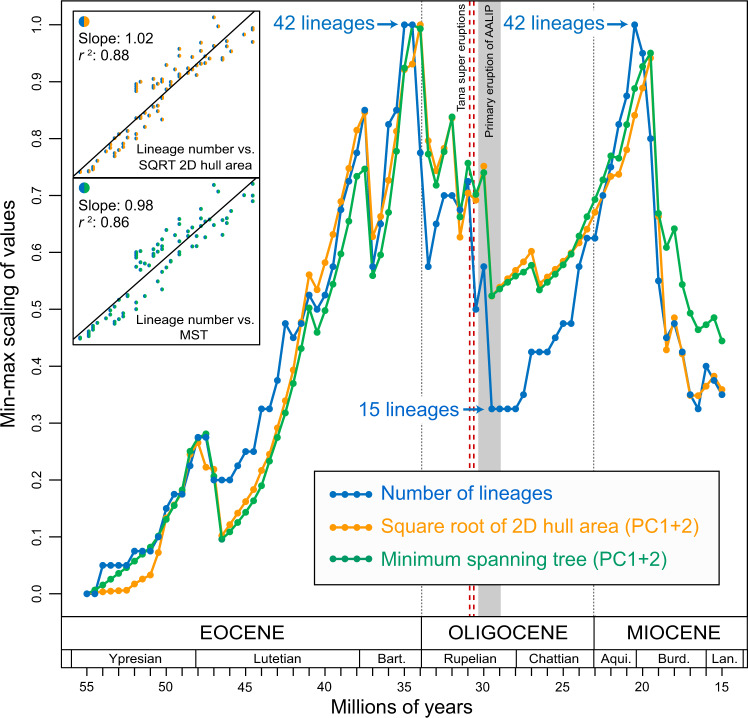
Lineage diversity and dental topographic disparity in Afro-Arabian primates and rodents from the early Eocene to the middle Miocene. The blue line is the LTT plot for the primate and rodent species for which M_2_ dental topographic metrics were calculated; the orange line shows temporal changes in the sum of the square roots of 2D hull areas for all four clades, with 2DHA calculated for each clade (Anomaluroidea, Anthropoidea, Hystricognathi, and Strepsirrhini) independently (sumSR2DHA); the green line shows temporal changes in the length of the minimum spanning tree (MST), calculated for each clade (Anomaluroidea, Anthropoidea, Hystricognathi, and Strepsirrhini) independently (sumMST). sumSR2DHA and sumMST show a tight correlation with lineage number, as shown by the *r*^2^ values in the insets (*n* = 80). Bart. = Bartonian, Aqui. = Aquitanian, Burd. = Burdigalian, Lan. = Langhian.

Among the disparity metrics used here, SR2DHA is the least impacted by outliers, and allows us to sum values for each of the four clades considered independently (Fig. 2). A comparison of lineage number with sumSR2DHA and sumMST between 55 and 15 Ma shows very similar temporal trends (slope 0.98–1.02; *r*^2^ = 0.86–0.88). Extinctions in the early Oligocene resulted in a substantial loss of sumSR2DHA and sumMST, with values at 30.5 Ma being about one half of peak late Eocene values. Over the subsequent 10 Myr, sumSR2DHA and sumMST gradually increase, reaching an early Miocene peak at ~95% of late Eocene values.

Rodents show a drop in dental topographic disparity (DTD) at the EOB regardless of which disparity metric is used (Supplementary Fig. 3), and regardless of whether Anomaluroidea and Hystricognathi are analyzed together (Supplementary Fig. 3), or independently (Supplementary Figs. 4 and 5). The drop in DTD is most precipitous among hystricognaths (Supplementary Fig. 4), whereas anomaluroids show a very gradual decline in DTD from the EOB into the early Miocene (Supplementary Fig. 3). Strepsirrhines show a small DTD dip from the latest Eocene to the EOB, but the overall trend through the Oligocene is for DTD to increase (Supplementary Figs. 6 and 7); however, this pattern is driven largely by the early Miocene stem daubentoniid *Propotto*, whose relatively flat molars are radically different from those of other Afro-Arabian strepsirrhines^22^. When *Propotto* is excluded, strepsirrhine DTD drops sharply just before the EOB, and then drops again at 32 Ma, with a slow subsequent increase that peaks in the early Miocene, but only to a maximum of half the DTD observed in the late Eocene (Supplementary Fig. 7). Anthropoidea is the only clade that shows an increase in DTD across the EOB, followed by large drops at 32 and 30 Ma, when DTD returns to its lowest level since the middle Eocene (i.e., shortly after Anthropoidea appeared in Afro-Arabia) (Supplementary Fig. 8). DTD then increases steadily through the late Oligocene into the early Miocene, but never reaches the peak seen at 32 Ma, even though lineage numbers are higher in the early Miocene than at any point in the Paleogene.

### Temporal shifts in dental topography and topographic morphospace

**Fig. 3:**
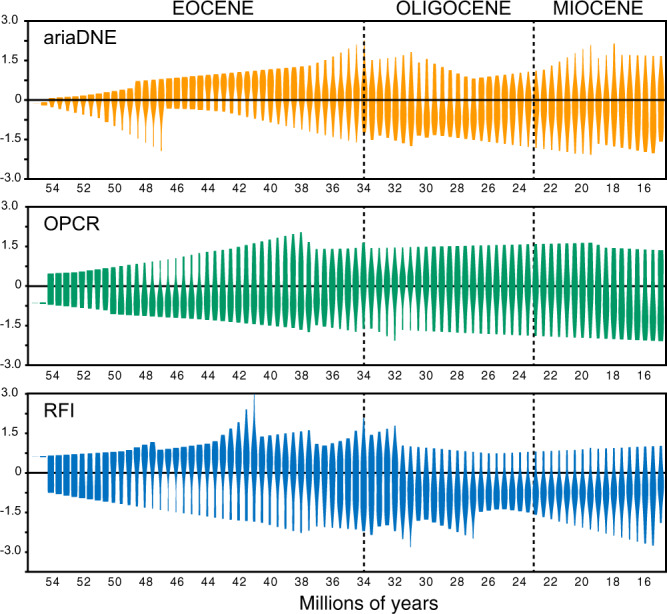
Long-term trends in primate and rodent dental topography through the Paleogene and early middle Miocene. Violin plots of all ancestral reconstructions for ariaDNE, OPCR, and RFI, collated at 0.5 Ma intervals.

When all of the ancestral states for primate and rodent DTMs are pooled, long-term shifts toward relatively high OPCR values and relatively low RFI and ariaDNE values are evident in the mid-Oligocene, following periods of increased variability for both metrics in the late Eocene (Fig. 3). RFI values remain relatively low through the early Miocene, while ariaDNE values show increased variability from the late Oligocene into the early Miocene.

**Fig. 4:**
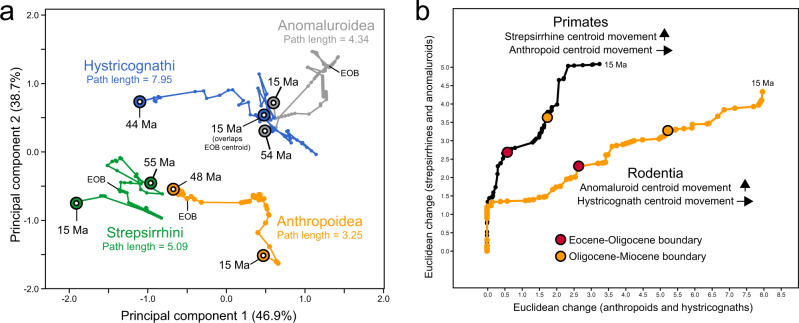
Movement of dental topographic morphospace centroids through time. **a** Plot of Euclidean movement of the centroid of each clade’s dental topographic morphospace from their inferred arrival time in Afro-Arabia (first divergence) to 15 Ma, and overall path length, along the first two principal components derived from PCA of the three dental topographic metrics (ariaDNE, OPCR, and RFI). **b** Comparison of Euclidean movement of morphospace centroids within each order (Primates and Rodentia), showing relative shifts through time; red and orange circles indicate the position of the Eocene-Oligocene and Oligocene-Miocene boundaries, respectively.

The centroid of the hystricognath dental topographic morphospace shifted farther and faster than that of any other clade considered here (movement of 0.28/Ma for hystricognaths versus 0.10–0.13/Ma for the other clades), and moved rapidly toward the ancestral anomaluroid morphospace during the middle and late Eocene (Fig. 4a). This shift suggests that early Afro-Arabian hystricognaths were evolving dental adaptations similar to those of early terrestrial stem anomaluroids such as zegdoumyids and nementchamyids, and so might have been exploiting similar dietary resources in terrestrial settings (Fig. 4b). The anomaluroid centroid shows a strong shift away from the group’s ancestral morphospace around 47–48 Ma, prior to the estimated ~44 Ma arrival time of Hystricognathi. The timing of this shift roughly coincides with the origin of crown Anomaluroidea^27^, members of which are either fully arboreal (Anomaluridae)^38^ or at least partially arboreal (Zenkerellidae)^39^, and so might reflect exploitation of novel dietary resources within an arboreal milieu. The evolution of arboreality presumably allowed crown anomaluroids to avoid direct competition with terrestrial hystricognaths for the remainder of the Cenozoic, but the same cannot be said of terrestrial or semi-terrestrial stem and basal crown anomaluroids, which were extirpated during the late Eocene interval when hystricognaths were undergoing a major adaptive radiation in Afro-Arabia.

Prior to the EOB, Afro-Arabian anthropoids show little movement of their dental topographic (DT) morphospace (total Eocene Euclidean path length of 0.59, Fig. 4a, b), whereas that of strepsirrhines shifts rather dramatically over the same interval (Eocene path length of 1.97) (Fig. 4a, b). This pattern suggests the possibility of competitive displacement of middle and late Eocene strepsirrhines by anthropoids within their shared arboreal habitats. However, in the early Oligocene, anthropoid feeding ecology undergoes a major change, as reflected by a strong shift in the position of that clade’s DT morphospace (i.e., away from that of strepsirrhines) between 32 and 30 Ma, likely due in part to the extinction of smaller-bodied anthropoid lineages (Fig. 4a; see also Kirk and Simons^40^). With an apparent release of ecological competition between anthropoids and strepsirrhines during the Oligocene, the latter then undergo a shift back toward the ancestral anthropoid DT morphospace from which they were displaced in the Eocene (Fig. 4a).

### Climatic and environmental context

Our data strongly suggest that Afro-Arabian primate and rodent clades lost most of their peak Eocene lineage diversity and DTD over the course of the first four million years of the Oligocene. All of the clades considered here (including Hyaenodonta) show a drop in lineage diversity within 500 Kyr of the onset of the early Oligocene global cooling event. There are no reliable high-resolution temperature proxies from continental Afro-Arabia across the EOT, so the possible influence of global cooling on this pattern of lineage loss is currently an open question; sea surface temperatures off the coast of Nigeria showed a ~2 °C drop across the EOT^41^, but estimates of mean annual temperatures based on poorly constrained equatorial terrestrial pollen records show no change during the same interval^11^. Afro-Arabia was largely isolated from northern continents at this time, so these extinctions cannot be attributed to competition with immigrant terrestrial taxa^12,42^. The same cannot be said of the middle Miocene diversity trough, by which time numerous Eurasian rodent clades had appeared in Africa^43^.

Anthropoidea, Hystricognathi, and Hyaenodonta all show a recovery after the EOB, followed by a second major loss of lineage diversity that bottoms out around 29.5–30 Ma. This time period is roughly coincident with land mammal age boundaries in North America (Whitneyan-Arikareean boundary, ~30 Ma)^44^, Europe (Suevian-Arvernian boundary, ~29.2 Ma)^44^, and South America (Tinguirirican-Deseadan boundary, ~30 Ma)^45^. All of these boundaries predate the Mid-Oligocene Glacial Interval (28–26.3), but are close to a major spike in atmospheric CO_2_ that peaks at ~29.5 Ma, followed by a steep drop through the rest of the Oligocene^46^.

The final extirpation of numerous Afro-Arabian mammal lineages around 30 Ma also roughly coincides with the onset of a series of major geological events that permanently and profoundly transformed the landscape of eastern Africa and the southwestern part of the Arabian peninsula—the uplift of the Ethiopian plateau^47^, volcanic super eruptions in Ethiopia at 31.1 and 30.8 Ma^48^, a massive outpouring of flood basalts of the Afro-Arabian Large Igneous Province (AALIP)^48^ (Fig. 2), and the opening of the Red Sea and Gulf of Aden^49^. Basalts of the AALIP are estimated to be ~900,000 km^3^ in volume^47^ and up to 3 km thick in some areas^48^. Unlike the effects of early Oligocene global cooling, which might have been buffered at lower latitudes, it is indisputable that these volcanic events must have had a devastating impact on the plant and animal communities that inhabited the AALIP, while also having a major influence on large-scale patterns of atmospheric moisture transport^50^ and water drainage^51^ in eastern Africa. It has recently been estimated that the release of sulfur and halogen molecules from the AALIP would have been comparable in volume to that which has been estimated for the Deccan and Siberian Traps^48^, both of which are temporally associated with mass extinction events (end-Cretaceous and end-Permian, respectively)^52^. Primates and rodents of early Oligocene age have been found around the periphery of the AALIP, indicating that this region was likely a major center of diversification for these clades. The only productive African fossil sites that fall within the mid-Oligocene diversity trough (the Chilga region of northwest Ethiopia^53^, which is geographically situated within the AALIP) have yielded numerous paenungulate species, but no rodents or primates, though the absence of small mammals at these localities might be due to taphonomic bias^53^. Nevertheless, the small number of late Oligocene sites that have been found in Afro-Arabia have thus far shown low primate and rodent diversity^54,55^, consistent with a scenario of early Oligocene extinction and late Oligocene reassembly of primate and rodent communities.

All of the foregoing suggests that explanations for the Oligocene extinctions that our analyses have revealed are likely complex and multifactorial, as there were global influences (such as cooling across the EOT, and changes in atmospheric CO_2_ levels) and local influences (such as AALIP volcanism) that could have caused major environmental changes in Afro-Arabia between ~34 and ~30 Ma. Any such scenarios must also take into account the varied evolutionary responses of other endemic clades during this turbulent interval—for instance, proboscideans diversified morphologically while embrithopods persisted into the late Oligocene relatively unchanged, at least dentally^56^; some hyracoid lineages that were present in the early Oligocene extended into the Miocene, but several went extinct in the early Oligocene^57^; and crown macroscelideans started to diversify in the Oligocene, but probably far to the southwest of the AALIP^58^. Crown bathyergoid rodents are estimated to have appeared in the mid-Oligocene in Afro-Arabia^59^, indicating the existence of selection pressures favoring fossoriality early in the Oligocene. It is tempting to suggest that hyaenodont extinctions in the early Oligocene might have followed from the loss of lineages that produced their preferred prey species; hyaenodont lineage diversity more closely tracks that of the relatively common anthropoids and hystricognaths (from 44 to 15 Ma; *r*^2^ = 0.81) than that of the relatively rare strepsirrhines and anomaluroids (from 54 to 15 Ma; *r*^2^ = 0.32). The lineage diversity and DTD patterns revealed here can be tested via similar analyses of these, and other, endemic African clades, but ultimately the more urgent need is improved paleontological sampling of the Afro-Arabian Oligocene. The environmental context for these pulses of extinction and diversification will only be clarified through a dedicated effort to extract paleotemperature estimates, stable isotopes, and paleobotanical data from diverse settings across Afro-Arabia that preserve terrestrial sediments of Oligocene age.

### Future applications

Our application of these phylogenetic and dental topographic methods to study long-term trends in lineage and dietary diversity is novel, and as DTMs are homology-free, this approach can be applied to any mammalian clade whose members retain functional molar teeth. Similar analyses of other mammalian clades evolving on other landmasses will therefore produce data that are directly comparable to those we present here, and we anticipate that such studies could provide significant new insights into the terrestrial mammalian response to global climate change through the Cenozoic.

## Methods

### Phylogenetics

The composite near-comprehensive tree of Afro-Arabian primates, anomaluroid rodents, and hystricognathous rodents combines information from multiple BTD analyses of character matrices published by Gunnell et al.^22^, Rasmussen et al.^60^, Dembo et al.^61^, DeSilva et al.^62^, Sallam and Seiffert^25^, and Marivaux et al.^16^. The tree is supplemented by molecular phylogenies of extant taxa^59,63^, and grafting of several extinct taxa that were not included in any of the aforementioned phylogenetic character matrices. All of the new BTD analyses employed the fossilized birth–death (=FBD) prior, flat beta priors for the *extinctionpr* and *fossilizationpr* parameters, exp(10) for the *speciationpr* parameter, and used the R code provided by Gunnell et al.^22^ to calculate an appropriate *clockratepr* prior (which uses as input the allcompat tree from a nonclock Bayesian analysis and the midpoint of each species’ age prior). Convergence of runs was assessed by tracking minimum effective sample sizes (=minESS) of all parameters, and average standard deviations of split frequencies (=ASDSF). Additional details of all phylogenetic analyses, and justifications for placements of grafted species, are available in the Supplementary Methods. The BTD analysis of hyaenodonts is based on a modified matrix that augments that of Borths and Stevens^64^ and includes a new hyaenodont genus and species from the terminal Eocene Fayum Locality 41. Phylogenetic data matrices, settings, and output are available in the Dryad repository associated with this study (10.5061/dryad.pc866t1nw).

### Calculation of lineage number through time

The composite time-scaled phylogeny includes 317 tips (198 fossil, 119 extant), all of which descend from unquestionably Afro-Arabian clades. The root age of this tree (representing the divergence between Primates and Rodentia) is ~76.16 Ma, based on the average of four different estimates for the origin of Euarchontoglires recovered by Springer et al.^65^. To quantify the number of lineages (i.e., branches) present in the tree at any given time, we used the function *ltt.plot.coords()* from the R package *ape*^66^ for continuous-age lineage through time (LTT) summarization.

### Phylogenetic randomization

With the set of 317 taxa, the function *rtree()* from the R package *ape* was used to create 10,000 random phylogenies. Branch lengths were stripped from these trees leaving only topological relationships. Each topology was then converted to a set of hard constraints using the function *createMrBayesConstraints()* from the R package *paleotree*^67^. Next, base R functions were used to create 10,000 Nexus character-taxon matrices, each containing the 317 taxa and 1,000 standard characters where all matrix cells were randomly coded as 0 or 2. After doing so, each set of topological constraints was paired with a unique character-taxon matrix. We used scripting with MrBayes v3.2.7^68^ to conduct Bayesian time-scaled phylogenetic analyses. Three constraint types were applied to each of these calls: (1) a fixed topology (see above), (2) fixed ages for taxa in the matrix (extracted from the starting composite tree), and (3) a fixed root age. MCMC settings for all analyses specified 1 run, 8 chains, and sampling for 1 M generations in increments of 1000. Fixed root ages were paired with approximately flat clock rate priors [norm(1,100)]. All characters were treated as ordered for branch length estimation from the random character matrices (i.e., binary standard characters treated as unordered will less effectively inform branch lengths in MrBayes analyses). Proposals that include Tau moves were disabled because these are incompatible with the application of hard topological constraints for the full tree. Posterior distributions were summarized using the option for 50% majority rule plus all compatible groups. The resultant set contains 10,000 trees where topology, branch lengths, and node ages are random—but tip times preserve the ages of their respective taxa. Lastly, LTT summarization was applied to each tree in the randomized set (see Fig. 1c). Both the composite tree and randomized tree-set are included in the Dryad repository associated with this study: 10.5061/dryad.pc866t1nw.

### Dental topographic data

A total of 329 second lower molar specimens of 134 species were scanned using a Nikon XTH 225 ST scanner (110–121 kVp, 116–120 mA, 0.013–0.034 voxel size), a Scanco vivaCT40 scanner (75kVp, 177 mA, 0.0125–0.035 voxel size), or a GE Phoenix Nanotom M scanner (90 kVp, 180 mA, 0.012 voxel size). Original fossil specimens or epoxy casts of fossil specimens were scanned. Surface files were created and oriented into occlusal view in Avizo version 8.0. Digital surface files were cropped at the lowest point of occlusal basin for calculation of ariaDNE and OPCR. All digital surfaces were also cropped along the base of the crown for the calculation of RFI. Minor deformities such as fine cracks in the enamel or small bubbles introduced during the molding process were manually removed in Geomagic. Surface files that had been cropped at the lowest point of the occlusal basin were simplified to 8000 triangles and smoothed for 30 iterations in Avizo (used for the calculation of ariaDNE and OPCR). Surfaces that had been cropped along the base of the crown were simplified to 10,000 triangles and smoothed for 30 iterations in Avizo (used for the calculation of RFI).

The variables ariaDNE (ε = 0.1), RFI, and OPCR were used to quantify dental shape. Dental topography was chosen to quantify dental shape as these GIS-based methods summarize the three-dimensional shape of a tooth in various functionally distinct metrics: complexity^34^, relief^36^, and bending energy^69^.

Complexity, or OPCR^34,70^ captures the number of “breakage sites”, or “patches” on a crown surface and is calculated as the OPCR. It is calculated by summing the number of “patches” that are formed by continuous points on the digital surface that face the same direction and have the same orientation^34,70^. The counting of patches is repeated eight times, every time rotating the crown surface slightly (5.625°). This “rotated” patch count decreases the error that is introduced by manually orientating the tooth into occlusal view. The OPCR value is the average of the eight Orientation Patch Counts. The more “patches”, the more “tools” the tooth has for breaking down food and thus the higher the complexity. OPCR was calculated for surfaces using MorphoTester^71^. Relief (or RFI) is measured as the ratio between 3D crown surface area and the 2D crown surface area^36^. RFI was calculated in Excel using the surface area and outline area output from MorphoTester following the same equation used by Boyer (2008):

1 $${{{{\mathrm{ln}}}}}\left(\frac{\sqrt{{{{{{\mathrm{Surface}}}}}}}{{{{{\mathrm{Area}}}}}}}{\sqrt{{{{{{\mathrm{Outline}}}}}}}{{{{{\mathrm{Area}}}}}}}\right).$$

Bending energy of a surface was calculated as the DNE of a surface, or ariaDNE^35,69^ and, simply put, captures the “curvedness” of the surface. ariaDNE was calculated using a the Matlab script provided by Shan et al.^35^ in Matlab version R2018a. The ε-value was set to 0.1, as we intended to capture large-scale features of molar shape but remained within the recommended ε-value range by Shan et al.^35^.

The topographic variables capture different aspects of the crown shape. Simply put, complexity reflects *number* of features on a surface, or “tools” (such as cusps, Evans et al., 2007)^34^, whereas curvature and relief reflect the *shape* of features (such as a sharp cusp compared to a blunt cusp, Bunn et al., 2011)^69^. However, in practice, all dental topographic variables are affected by both the *number* and *shape* of features of teeth, but they are affected to different degrees (see Winchester (2016)^71^ for a further discussion of how these different topographic variables correlate with each other or are affected by simulated differences in 3D shapes).

Dental topographic values were averaged per species. On average, each species was represented by 2.5 specimens. Specimens with extreme wear were excluded following criteria outlined by Allen et al.^72^. A list of all specimens used in the DTM analyses is provided in the Dryad repository associated with this study: 10.5061/dryad.pc866t1nw.

### Principal components analysis

A PCA was applied to the species averages of the complete sample using the three variables ln(OPCR), RFI, and ariaDNE. The natural log of the OPCR values was taken as count-data, and cannot be treated as continuous data^73^. The PCA was performed in R using the *prcomp*() function from the *stats* package and setting “scale=TRUE”, which standardizes all variables prior to the analysis. PC1 captured 46.9% of the variation, PC2 38.7%, and PC3 14.4%.

### Ancestral state reconstruction

Ancestral state reconstruction (ASR) analyses were run independently for the three DTMs (ariaDNE, OPCR, and RFI), and jointly for the first two PCs from the PCA of these metrics. Again, together PC1 and PC2 explain ~85.5% of the observed variation. Before ASR, the data for each DTM were *z*-transformed (i.e., centered and scaled with means and standard deviations). In total, the trait data include 134 euarchontan taxa—47 anthropoid primates (43 fossil, 4 extant), 24 strepsirrhine primates (19 fossil, 5 extant), 47 hystricognath rodents (43 fossil, 4 extant), and 16 anomaluroid rodents (12 fossil, 4 extant). A time-scaled tree that corresponds to these taxa was created by pruning tips from the larger composite phylogeny leaving only terminals with data affirmative DTMs.

We tested the application of multiple Brownian motion (BM) rate matrices to explore if DTM evolution among the four focus clades (anthropoids, strepsirrhines, hystricognaths, and anomaluroids) could be better modeled in a multivariate context. To do so, we defined each group as its own “regime” and then created all possible combinations thereof. These combinations include: one version with a single BM rate (=regime), seven versions with two rates, six versions with three rates, and one version with four rates. For the two-PC trait set, we also created three variants for each version—a variant where the two trait rate matrices are estimated independently (i.e., unique variances and shared covariance; unconstrained), a variant where the rate (=sigma) for each PC is estimated independently but covariance is disallowed (i.e., a “diagonal” constraint), and a variant where the two PCs share a single rate (i.e., an “equal” constraint, without covariance). In total, this strategy produced 15 combinatorial versions for each DTM data set and 45 combinatorial versions/variants for the two-PC data set.

The function *mvBM()* (multivariate Brownian motion) from the R package *mvMORPH*^74^ was used to obtain AICc decision-criterion scores for all combinatorial versions (and their variants). The AICc calculation includes penalty terms for the invocation of additional model parameters which allows for optimal model choice, without overfitting. For each of the four trait-sets (ariaDNE, OPCR, RFI, and two-PC), model tests on the version-suites were repeated ten times and the models producing the lowest AICc scores were selected as preferred.

For the tests using two-PC trait data, all ten replicates identified the best-fit model as including two BM rate matrices (1 = anomaluroids, 2 = anthropoids + strepsirrhines + hystricognaths) treated as unconstrained (i.e., unique rates for each PC with shared covariance). For ariaDNE, the ten replicates produced mixed results, with a near tie between two models—one that includes four BM rates (i.e., one rate per clade) and one that includes three BM rates (1 = hystricognaths, 2 = anomaluroids, 3 = anthropoids + strepsirrhines). The ten OPCR model tests also produced mixed results with optimal AICc scores corresponding to a three-rate model (1 = anthropoids, 2 = hystricognaths, 3 = strepsirrhines + anomaluroids) and a two-rate model (1 = anthropoids + hystricognaths, 2 = strepsirrhines + anomaluroids). For RFI, the ten tests found two best-fit models which incorporate four rates (i.e., one rate per clade) and three rates (1 = hystricognaths, 2 = anomaluroids, 3 = anthropoids + strepsirrhines). Using the phylogenetic tree, the four trait data sets, and these model test results, we used the function *estim()* from the R package *mvMORPH* to estimate continuous-trait values for all internal tree nodes. For each of the four trait data sets, we repeated these ASR estimations ten times (i.e., once per model test result). Final nodal estimates were calculated as the arithmetic means from the ten rounds of ASR.

### Time-slice trait value interpolation

Within a phylogenetic tree, each edge (i.e., branch) is defined by (and connected by) a unique pair of nodes, the parent and child. Tree tips are considered terminal tree nodes for which there are no descending children. For a time-scaled phylogenetic tree, each parent–child pair has an estimated start and end age. If the same tree has been used in an ASR analysis, each parent–child pair will also be associated with start and end values for the reconstructed trait. Thus, a simple linear rate of change can be calculated for each tree edge and the trait’s value can be interpolated at all positions along any edge in the tree.

Further, at any given age contained in a time-scaled tree, all edges (i.e., lineages) that cross that time-slice can be counted. This is discrete-age lineage through time (LTT) summarization. Similarly, at any given age we can collect a distribution of interpolated trait values from the set of edges that cross the time-slice. If this strategy is repeated at small age increments, the distributions of trait values from multiple time-slices can characterize (nearly continuously) the evolution of variation in the trait through time.

We produced novel R code to implement time-slice trait value interpolation. Input for the code includes a time-scaled phylogenetic tree (ours is discussed above), observed trait values for tree tips (i.e., input for the ASR analyses), and estimated trait values for internal tree nodes (i.e., output from the ASR analyses). The R code also includes an input argument that specifies age increment sampling. We selected increments at 0.5 Myr which samples our tree at 153 time-slices ranging from present (0 Ma) to the late Cretaceous (76 Ma). The output generated by our code is a simple table where column names are time-slice ages and row names are tree edge numbers (following the standard edge numbering scheme used for class “phylo” objects in R). Table cells contain the interpolated values of the trait for tree edges at their corresponding time-slice. Where edges do not cross a time-slice, table cells are populated with not applicable (NA). We applied this code to all three DTMs and both PCs.

### Time-slice summarization and calculation of disparity through time

The phylogeny used for ASR analyses contains 12 tree edges that cannot (firmly) be associated with residency on the Afro-Arabian landmass. These edges are the stem branches leading to the clades (1) primates, (2) anthropoids, (3) strepsirrhines, (4) rodents, (5) hystricognaths, and (6) anomaluroids. Further, the branches (7,8) leading to the terminals for *Lemur catta* and *Nycticebus coucang* (extant taxa from Madagascar and Indonesia, respectively) almost certainly do not represent occupancy on the African continent. We also identified the stem branches (9,10,11,12) associated with the clades (a) Azibiidae + Djebelemuridae, (b) Adapidae, and (c) *Nosmips* + crown Strepsirrhini as possibly non-African. With the intent to limit our characterizations to lineages that can be confidently referred to Afro-Arabia, we elected to exclude these tree edges from the results—prior to summarization—by removing their corresponding rows from the trait value interpolation tables. We assigned each of the remaining tree edges to one of the focus clades creating four primary edge subsets (anthropoids, strepsirrhines, hystricognaths, and anomaluroids), and then regrouped two of these subsets into a “rodents” set, two into a “primates” set, and all four back into an “all clades” set. One additional exploratory subset removed the *Propotto* edge from the strepsirrhine group.

We used the “all clades” edge set to summarize ariaDNE, OPCR, and RFI for time-slice samples. These distributions were characterized using kernel density estimation (KDE) and plotted as violins (Fig. 3). Summarization of PC1 + PC2 was applied independently to the four focus subsets (i.e., clades), to the strepsirrhine (less *Propotto*) subset, to the recombined “rodents” set, and to the recombined “primates” set (Supplementary Figs. 3–8). Each of these summarizations begins with the oldest data affirmative time-slice and ends at present day (0 Ma). We calculated four measures of variation for each time-slice: SoV, SoR, SR2DHA, and MST. SoV is simply the variance of PC1 added to the variance of PC2. Similarly, SoR is the range of PC1 (difference between max and min) added to the range of PC2. SR2DHA treats PC1 + PC2 coordinates in each time-slice distribution as an *XY* point cloud and a hull is estimated around the points. The area of the resulting polygon quantifies the distribution’s occupancy of morphospace. To find SR2DHA, we used the function *concaveman()* from the R package *concaveman*^75^ with the *concavity* argument set to 10,000—a convex setting for our data which ranges approximately from −3 to 3 on both axes. Then, area was calculated using the function *Polygon* from the R package *sp*^76^, followed by square root transformation. MST point associations were found using the function *mst*() from the R package *ape*. The lengths of each of these linkages were calculated as standard Euclidean distance and all lengths in the network were summed to yield the MST metric.

Additionally, we computed per time-slice point cloud centroids (where *X* and *Y* are the arithmetic means of PC1 and PC2, respectively) and calculated the Euclidean distance between point cloud centroids of adjacent time-slices (Fig. 4a, b). Finally, we counted the number of lineages present in each time-slice sample. As a total measure of PC1 + PC2 variation, we summed the independent SR2DHAs of the four focus clades at each time-slice (Fig. 2).

Original data, model tests, ASR results, interpolation tables, and summarizations are available in the Dryad repository associated with this study (10.5061/dryad.pc866t1nw). We also provide all novel R scripts (and functions) as well as a step-by-step R workflow that reproduces this portion of the research.

### Statistics and reproducibility

The principal components analysis was performed in R using the *prcomp*() function from the *stats* package and setting “scale=TRUE”, which standardizes all variables prior to the analysis. Correlation analyses were performed in R. All input files for phylogenetic analyses, ancestral state reconstructions, time-slice trait value interpolation, and time-slice summarization and calculation of disparity through time are available on the Dryad Digital Repository (10.5061/dryad.pc866t1nw) and include all of the settings required to reproduce our results.

### Reporting summary

Further information on research design is available in the Nature Research Reporting Summary linked to this article.

## Supplementary information

Supplementary information.

Reporting summary.

## Data Availability

Input data files, settings, code and results from phylogenetic, ASR, and disparity analyses are available on the Dryad Digital Repository (10.5061/dryad.pc866t1nw)^77^. Digital surface models for all of the figured fossil specimens are available on MorphoSource (www.morphosource.org), except those housed at the National Museums of Kenya.

## Code availability

All novel R code (R version 4.0.2) used in this study is available on the Dryad Digital Data Repository (10.5061/dryad.pc866t1nw).
