# Supplementary material for: Widespread loss of mammalian lineage and dietary diversity in the early Oligocene of Afro-Arabia
Source: Commun Biol. 2021 Oct 7;4:1172. doi: 10.1038/s42003-021-02707-9 (PMC8497553; doi:10.1038/s42003-021-02707-9)
Supplement: Supplementary file 1 — Supplementary information. [file 42003_2021_2707_MOESM1_ESM.pdf]

Supplementary information for "Widespread loss of mammalian lineage and dietary diversity in the early Oligocene of Afro-Arabia"

Dorien de Vries[1,2], Steven Heritage[2,3], Matthew R. Borths[3], Hesham M. Sallam[4,5], Erik R. Seiffert[3,6,7]

[1]Ecosystems and Environment Research Centre, School of Science, Engineering and Environment, University of Salford, Manchester, U.K.

[2]Interdepartmental Doctoral Program in Anthropological Sciences, Stony Brook University, Stony Brook, New York, 11794, U.S.A.

[3]Duke Lemur Center Museum of Natural History, Durham, North Carolina, 27705, U.S.A.

[4]Mansoura University Vertebrate Paleontology, Department of Geology, Faculty of Science, Mansoura, Egypt

[5]Institute of Global Health and Human Ecology (I-GHHE), School of Sciences and Engineering, American University in Cairo, New Cairo, Egypt

[6]Department of Integrative Anatomical Sciences, Keck School of Medicine of USC, University of Southern California, Los Angeles, California, 90033, U.S.A.

[7]Department of Mammalogy, Natural History Museum of Los Angeles County, Los Angeles, California, 90007, U.S.A.

Supplementary Methods

Construction of the near-comprehensive composite tree

The near-comprehensive composite tree of living and extinct Afro-Arabian anomaluroid rodents, hystricognath rodents, anthropoid primates, and strepsirrhine primates is based largely on the results of individual Bayesian tip-dating (BTD) analyses of morphological and combined (molecular/morphological) matrices that were manually combined into a single tree. The tree was further expanded by including time-scaled molecular phylogenies of extant species, details of which are given below. In addition, some fossil species that were not included in any of the morphological matrices were grafted onto the tree in positions that we considered reasonable and justifiable given available evidence; we provide justifications for all of these decisions below.

As the primary time period of interest for our study ranges from the Eocene into the middle Miocene, we were most focused on ensuring that that time period was comprehensively sampled. For this reason, we did not attempt to place various Plio-Pleistocene species (for instance fossil cercopithecoids and galagids from that time period that have not been placed in phylogenetic context) into our composite tree. Including such species would be expected to further elevate the lineage diversification curve during the Plio-Pleistocene, but their inclusion would in no way impact the results or conclusions of our analysis. For our comparisons of disparity in dental topographic metrics (DTMs) and lineage diversity, we simply removed all branches for which we lacked dental topographic data. As noted in the main text, lineage counts between 55 and 15 Ma (taken at 0.5 Ma intervals) in the reduced sample on which we calculated DTMs closely track lineage counts in the composite tree (r^2=0.97).

Consensus trees that have been calculated in MrBayes are output with tip and node ages that include up to 7 significant digits. Manual grafting of multiple time-scaled trees with such precise values inevitably leads some extant tip ages to not line up at exactly 0 Ma. As these non-0 values of extant tips produce artificial extinction signals in the youngest bin of the lineage-through-time (LTT) plots, we used an R script written by S.H. that ensures that all extant tips are perfectly flush and line up at 0 Ma. The function achieves this goal by slightly adjusting other nodes in the tree, leading to differences from node and tip ages of input trees that are less than 0.02 Ma. This explains why (for instance) tip and node ages in the original "allcompat" tree of Gunnell et al. [1] are very slightly different from those in our composite tree. The R script for this function is available in the Dryad repository associated with this study (https://doi.org/10.5061/dryad.pc866t1nw).

Strepsirrhines, tarsiers, and early anthropoids including basal stem catarrhines. For all primate clades aside from crown and near-crown Catarrhini, we used the time-scaled tree of Gunnell et al. [1], which is based on a BTD analysis of combined molecular [61,119 positions; 69 nuclear and 10 mitochondrial gene segments from Springer et al. [2]] and morphological data (395 characters). Phylogenetic methods were described in Gunnell et al.[1], and the original input and output files can be found at https://doi.org/10.5061/dryad.gb182. Of the 57 primate species in the comprehensive tree that are outside of crown or near-crown Catarrhini, 34 are sampled in the matrix of Gunnell et al. [1], and provide a robust backbone onto which unsampled species could be grafted. In most cases, grafted species represent species of sampled genera that were not included in Gunnell et al. [1] because they are highly incomplete.

Our justifications for the species or clades grafted onto the backbone provided by the tree of Gunnell et al.[1] are as follows:

- The latest Paleocene primate *Altiatlasius koulchii* from the Ouarzazate Basin of Morocco is of uncertain phylogenetic position; anthropoid affinities have most commonly been discussed (e.g., Godinot [3]; Beard & Wang [4]), but placement among plesiadapiforms has also been proposed [5]. We conservatively placed it in an unresolved position at the base of crown primates.
- Early Oligocene *Afrotarsius chatrathi* [6] was placed as the sister taxon of late Eocene *Afrotarsius libycus* [7]. The tip of the "*Afrotarsius* spp." OTU employed by Gunnell et al. [1], which included character data from both *A. chathrathi* and *A. libycus*, was placed at 35.59 Ma in their BTD analysis. This age is highly consistent with the age estimates for hystricognath rodents from the Dur at-Talah (DT) "locality 1" (DT-Loc-1) that yielded *Afrotarsius libycus* 36.2 Ma [8], and so was used as the tip age for *A. libycus*. The tip of the *A. chatrathi* lineage was placed at the mean (29.6 Ma) of all calculated tip ages (i.e., in the tree of Gunnell et al. [1]) for sampled taxa from Quarry M in the Jebel Qatrani Formation of Egypt, the site where the single specimen of *A. chathrathi* was found [6].
- The early Oligocene strepsirrhine *Omanodon* from Taqah, Oman [9] appears to be more closely aligned with "*Anchomomys*" *milleri* from the late Eocene of Egypt than with any other living or extinct strepsirrhines. We accordingly placed *Omanodon* as the sister taxon of "*A.*" *milleri* and assigned its tip age as the average of the tip ages of the other primate species from Taqah (the "Taqah propliopithecid" and *Oligopithecus rogeri*) that were included in the matrix of Gunnell et al. [1].
- Godinot et al. [10] described a new caenopithecine adapiform, *Namadapis interdictus*, from the middle Eocene Black Crow locality in the Sperrgebiet region of Namibia. Given the much older age and generally plesiomorphic morphology of *Namadapis* relative to other African caenopithecines (*Afradapis*, *Aframonius*, *Masradapis*), we placed it as the sister taxon of a clade containing those younger species, separated from that node by a 1 Ma-long branch. For its tip age, we used the age calculated for the anomaluroid rodent *Zegdoumys namibiensis* (i.e., 44.5 Ma; Lutetian age prior of 41.2-47.8 Ma) in our BTD analysis of anomaluroids (see below).
- *Karanisia arenula* from the late Eocene of Libya [7] was placed as the sister taxon of its congeneric species *Karanisia clarki* [11]. The 36.2 Ma age for DT-Loc-1 is derived from BTD analysis of hystricognath rodents [8], and the older taxon *Karanisia clarki* was assigned a 1 Ma-long branch.
- There is currently no consensus on the phylogenetic placement of the early Miocene lorisiform genus *Progalago* relative to Lorisidae and Galagidae [12,13]. We conservatively placed *Progalago* near the base of crown Lorisiformes, separated by a short 0.2 Ma from the crown lorisiform node. *Progalago dorae* and *Progalago songhorensis* were placed as sister taxa, each with 1 Ma-long branches. Note that this conservative arrangement requires the presence of a ghost lineage extending through the entire Oligocene and into the late Eocene, and so is biased against the hypothesis of an Oligocene extinction; an alternative placement of *Progalago* species as stem galagids or stem lorisids of more recent (late Oligocene or early Miocene) origin would have further reduced the lineage diversity curve during the early Oligocene.
- The unnamed lorisid from Fort Ternan, Kenya, is approximately 13.7 Ma in age [14]. Harrison [13] suggested that the taxon could be specifically related to *Perodicticus* among the Lorisidae, but this relationship cannot be accommodated by the ~12.5 Ma divergence between *Arctocebus* and *Perodicticus* in our tree; the most reasonable alternative placement given

these constraints is as a sister taxon of the *Arctocebus-Perodicticus* clade. The better-known genus *Mioeuoticus* also cannot be placed within the *Arctocebus-Perodicticus* clade due to the aforementioned age discrepancy. We placed all members of *Mioeuoticus* in a clade that is the sister taxon of *Arctocebus-Perodicticus* plus the Fort Ternan lorisid, with the oldest species *Mioeuoticus bishopi* (given the same age as *Paranomalurus bishopi* — 18.9 Ma as determined by BTD analysis of anomaluroids) placed as the most basal taxon, and *Mioeuoticus kichotoi* (14.7 Ma [15]) and *Mioeuoticus shipmani* (mean of all tip ages for taxa from Rusinga, 17.6 Ma, as determined by BTD analysis) situated as sister taxa.

- The early-middle Miocene genus *Komba* was represented in the matrix of Gunnell et al. [1] by an OTU "*Komba* spp." that primarily included scores from *Komba robustus*. For purposes of the composite tree we placed the younger *Komba* species *walkeri* [13] and *winamensis* [16] as sister taxa, with the older species *minor* and *robustus* in an effective trichotomy with that clade (with latter species separated by a short 0.2 Ma-long branch).
- The phylogenetic relationships of galagids are based on the molecular phylogenetic and dating analysis of Springer et al. [2] with autocorrelated rates and hard bounds. The same molecular dataset was used in the combined molecular-morphological BTD analysis of Gunnell et al. [1], but that analysis returned divergence dates within Galagidae that were on average 63.2% younger than those of Springer et al.'s tree with hard bounds. To maintain internal consistency, we shortened all of the branch lengths within crown Galagidae by multiplying them by 0.632.
- The primate *Amamria tunisiensis* from the Eocene of Tunisia [17] is only represented by a single upper molar, and the taxon is of uncertain phylogenetic position. Though we consider other phylogenetic placements to be possible, for purposes of this analysis we follow Marivaux et al. [17] in placing this taxon as a primitive anthropoid in an unresolved position relative to parapithecoids and crown anthropoids, with a tip date equal to that of the estimate of 39.5 Ma that the BTD analysis of Sallam and Seiffert [18] returned for the rodent from Djebel el Kébar, "*Protophiomys*" *tunisiensis* [19].
- *Biretia piveteaui*, from the middle or late Eocene of Algeria [20] was placed in an effective trichotomy (separated by an internode of 0.2 Ma) with *Biretia* species that were included in the BTD analysis of Gunnell et al. [1].
- *Qatrania fleaglei*, from Quarry M in the Jebel Qatrani Formation of Egypt [21], was placed as the sister taxon of *Qatrania wingi*, its older relative from Quarry E in the same formation. *Qatrania wingi* was assigned a 1 Ma-long terminal branch and *Q. fleaglei*'s tip was placed at 29.6 Ma, the mean age for all other species from Quarry M.
- We follow Ducrocq et al. [22], who suggested that *Lokonepithecus manai* from the Oligocene of Turkana, northern Kenya, was best placed in an unresolved position with respect to *Apidium* and *Parapithecus*. *L. manai*'s tip date (31.9 Ma) is based on the age assigned to the Lokone hystricognath *Turkanamys hexalophus* in the BTD analysis of Sallam and Seiffert [8].
- *Apidium zuetina* from Zallah Oasis, Libya, was placed as the sister taxon of *Apidium phiomense* following the parsimony analysis of Beard et al. [23]. The tip age for *A. zuetina* was based on the mean value for hystricognaths from the correlative Quarry G/V level in the Jebel Qatrani Formation (30.8 Ma [8]) and was assigned a terminal branch length of 1 Ma, leaving a short (488 kyr) branch separating the *phiomense-zuetina* clade from its shared node with *Apidium moustafai*.
- *Apidium bowni* was placed as the sister species of *Apidium phiomense*, *Apidium zuetina*, and *Apidium moustafai* (see also [23,24]), with its tip age based on the 30.8 Ma estimate derived

from BTD analysis of hystricognaths from the same locality [8]. The internode separating *A. bowni* from the *A. moustafai-phiomense-zuetina* clade was set as 1 Ma long.

- *Talahpithecus parvus* from the late Eocene of Libya was placed in the family Oligopithecidae by Jaeger et al. [7], a hypothesis that we also consider to be the best-supported based on the limited material available for the species. We place *T. parvus* as the most basal oligopithecid, outside of a clade containing *Catopithecus* and *Oligopithecus*, separated by an internode of 1 Ma. The 36.2 Ma age for DT-Loc-1 is derived from BTD analysis of that site's hystricognath rodents [8].
- The unnamed oligopithecid from Quarry M in the Jebel Qatrani Formation of Egypt [25] is only known from an M_1 implanted in partial hemi-mandible, and so is difficult to place relative to better known oligopithecid species. We place it in a trichotomy with *Catopithecus* and *Oligopithecus*, and place its tip at 29.6 Ma, the mean age for all other species from Quarry M.
- *Propliopithecus ankeli* has previously been synonymized with the "Taqah propliopithecid" [26], the latter of which was included in the matrix of Gunnell et al. [1]. We accordingly place *P. ankeli* as the sister taxon of the Taqah propliopithecid,
- The taxonomy and phylogenetic position of the poorly known early catarrhine *Moeripithecus markgrafi* is not clear; some [27] consider it to be the same taxon as the "Taqah propliopithecid" from the early Oligocene of Oman, while others consider *M. markgrafi* to be valid [28]. Both *M. markgrafi* and *Parapithecus fraasi* are of unknown provenance in the Jebel Qatrani Formation, having been collected without field notes by Richard Markgraf in the early part of the 20th century. Based on the taxon's transitional characters, we place *M. markgrafi* at the base of the clade containing propliopithecids and all later catarrhines, with its tip date being the same as *Parapithecus fraasi* (30.52 Ma).
- *Propliopithecus haeckeli* is most similar to *Propliopithecus chirobates* among propliopithecids [28]. We placed *P. haeckeli* as the sister taxon of *P. chirobates*, with the former's tip age based on the age of *Parapithecus fraasi* as estimated by BTD analysis (*P. fraasi* being another species that was discovered by Richard Markgraf in the early part of the 20th century, with no notes on the geographic or stratigraphic position of his localities; Markgraf's primate discoveries are of unknown provenance but are not inconsistent with collection from a single locality).

Crown catarrhines and near-crown stem catarrhines, aside from Hominina. Relationships of late Oligocene and early-middle Miocene catarrhines were based on a BTD analysis of the matrix of Rasmussen et al. [29]. We scored late Oligocene *Kamoyapithecus* [30], *Rukwapithecus* [31], and *Saadanius* [32], and early Miocene *Noropithecus* for upper dental characters [33], following Stevens et al. [31] and based on new observations. We also added the scores of Rossie and MacLatchy [34] for early Miocene *Kalepithecus* and *Limnopithecus*, and middle Miocene *Pliopithecus*, which were excluded from the matrix of Rasmussen et al. [29].

Several hard constraints were employed to ensure that the resulting allcompat tree could be easily grafted onto that of Gunnell et al. [1]. These constrains enforced the monophyly of 1) all catarrhines aside from *Aegyptopithecus* and *Catopithecus*; 2) all catarrhines aside from *Catopithecus*; 3) all catarrhines (including *Catopithecus*); 4) all platyrrhines; 5) *Saimiri-Cebus* to the exclusion of *Aotus*; 6) *Gorilla-Pan* to the exclusion of *Pongo*; 7) *Gorilla-Pan-Pongo* to the exclusion of all other species; 8) *Cercopithecus-Macaca* to the exclusion of *Presbytis*; 8) *Cercopithecus-Macaca-Presbytis* to the exclusion of all other species. The divergence of

Catopithecus from other catarrhines was constrained to be 40.09 Ma, and the divergence of *Aegyptopithecus* from other catarrhines was constrained to be 34.02 Ma, as in the tree of Gunnell et al. [1]. Divergence dates among extant taxa in the matrix were set to match the mean divergence dates reported in Table 1 of Springer et al. [2]. The fossilized birth-death prior was implemented, and the sampleprob prior was set as 0.038, based on the 184 catarrhine species reported by Fleagle and Seiffert [35]; we used flat beta priors for the extinctionpr and fossilizationpr parameters and exp(10) for the speciationpr parameter. The clockratepr parameter was set as lognormal(-2.533, 0.852), based on the results of the R script of Gunnell et al. [1], which used as input the allcompat tree from a nonclock Bayesian analysis and the midpoint of each species' age prior. The analysis was run for 50 million generations, with four chains and a temperature of 0.02, and the first 25% of the sample was discarded as burn-in before computing the consensus tree. The average standard deviation of split frequencies (ASDSF) in the final generation was 0.003, and the lowest effective sample size (ESS) value was 5132.6; both diagnostics provide strong support for convergence.

The primary topological differences between the resulting allcompat consensus and the parsimony-based strict consensus of Rasmussen et al. [29] are 1) placement of *Nsungwepithecus* as the most basal stem cercopithecoid, in place of earliest Miocene *Alophe*; 2) placement of *Prohylobates* and *Noropithecus* as consecutive sister taxa of *Victoriapithecus* + crown Cercopithecoidea, rather than *Noropithecus* being the sister taxon of *Victoriapithecus*; 3) placement of *Micropithecus clarki* as a relatively crownward stem hominoid, rather than as a stem catarrhine; 4) placement of *Simiolus* as the sister taxon of *Dendropithecus*, and both placed together as the sister clade of *Limnopithecus* — which together form one branch along the hominoid stem lineage — rather than being placed as sister taxa of nyanzapithecines; 5) *Turkanapithecus* falling within *Nyanzapithecus*, rather than being placed as the sister taxon of *Rangwapithecus* + *Nyanzapithecus*; 6) placement of *Ekembo* as the sister taxon of *Kalepithecus*, rather than as the sister taxon of *Proconsul*; 7) placement of *Morotopithecus* alone as the most crownward stem hominoid, rather than *Afropithecus* + *Morotopithecus*, with *Equatorius* intervening between the latter two taxa along the hominoid stem lineage.

Our justifications for the species or clades grafted onto the backbone provided by the revised analysis of Rasmussen et al. [29] are as follows:

- The early Miocene small "apes" *Iriripithecus*, *Karamojapithecus*, and *Kogolepithecus* described by Pickford et al. [36] were hypothesized by those authors to be sister taxa of *Lomorupithecus*. However, their conception of *Lomorupithecus* differs from that of Rossie and MacLatchy, who erected the genus; Rossie and MacLatchy considered *Lomorupithecus* to be a possible pliopithecoid with no relationship to *Limnopithecus*, whereas Pickford et al. transferred *Limnopithecus evansi* into the genus *Lomorupithecus*. Pickford et al.'s placement of *Limnopithecus evansi* into *Lomorupithecus* has since been criticized by Cote et al. [37], who considered both *Lomorupithecus harrisoni* and *Limnopithecus evansi* to be valid taxa. As reconciliation of this disagreement is beyond the scope of this contribution, we decided to place *Iriripithecus*, *Karamojapithecus*, and *Kogolepithecus* as sister taxa of *Limnopithecus evansi* (i.e., most of what makes up the hypodigm of "*Lomorupithecus evansi*"), assuming that this arrangement most faithfully comports with Pickford et al.'s proposed phylogenetic placement.

- *Simiolus minutus* from the lower part of the Ngorora Formation (Kenya) [38] was placed as the sister taxon of early Miocene *Simiolus enjiessi*, with a tip date midway between the upper and lower bounds for the age of the site (12.56-12.3 Ma).
- Additional species of *Micropithecus* (*leakeyorum* and *songhorensis*) were placed in an effective trichotomy (intervening short branch of 0.2 Ma) with *Micropithecus clarki*; the branch for the oldest species (*M. songhorensis*) was set as 1 Ma, thereby determining branch lengths for the other species.
- For the placement of *Kenyapithecus wickeri* and *Nyanzapithecus alesi*, which were not included in the analysis of Rasmussen et al. [29], we ran a BTD analysis of the matrix published by Nengo et al. [39]. In this BTD analysis *N. alesi* was situated as the sister taxon of a clade containing *Nyanzapithecus harrisoni* and *Nyanzapithecus pickfordi*, with a divergence from those taxa at 17.29 Ma, which cannot be accommodated by the 17.5 Ma divergence between those taxa in the BTD analysis of Rasmussen et al. We instead connected the *N. alesi* branch midway along the branch between *N. harrisoni*-*N. pickfordi* and *Turkanapithecus* (at 18.75 Ma). *Kenyapithecus* was placed as a stem pongine, and thus more closely related to *Pan-Gorilla* than to *Morotopithecus*, the taxon closest to crown Hominoidea in the BTD analysis of Rasmussen et al. [29]. We therefore grafted *Kenyapithecus* onto the tree in this position, with the same divergence date as recovered in the BTD analysis of Nengo et al. [39]. The input and output files of this analysis are available in the Dryad repository for this study (https://doi.org/10.5061/dryad.pc866t1nw).
- We placed the middle Miocene hominoid *Otavipithecus namibiensis* as the sister taxon of *Afropithecus turkanensis* following Singleton [40], and assigned it a tip age of 13 Ma.
- The ~15 Ma hominoid *Nacholapithecus kerioi* [41] was placed in an effective trichotomy with *Kenyapithecus* and later hominoids, with a short 0.2 Ma-long branch separating it from that clade.
- The late Miocene hominoids *Chororapithecus abyssinicus* [42] and *Nakalipithecus nakayamai* [43] have both been proposed as possible members of the *Gorilla-Pan-Homo* clade, but given the mean divergence dates among extant taxa recovered by Springer et al.[2], these fossil hominoids could only be accommodated as stem members of the *Gorilla-Pan-Homo* clade. We arbitrarily placed the slightly younger *Nakalipithecus* (tip age of 9.85 Ma) as being more closely related to *Gorilla-Pan-Homo* than *Chororapithecus* (tip age of 10.4 Ma).
- The stem cercopithecoid *Zaltanpithecus simonsi* from the Jebel Zaltan locality in Libya is of uncertain age; Werdelin [44] suggested that the site could be as old as 19 Ma and as young as 14 Ma. We take the midpoint of this estimate for the tip age of *Z. simonsi* (16.5 Ma). We place the taxon along the cercopithecoid stem, between *Noropithecus* and *Prohylobates*.
- Given the divergence dates of Springer et al. [2], the ~9.5 Ma *Microcolobus tugenensis* could only be placed as a stem colobine; it was assigned a 1 Ma-long branch that attached to the stem lineage of Colobinae.
- The divergence dates between *Pan troglodytes* and *Pan paniscus*, and between *Gorilla gorilla* and *Gorilla beringei*, were based on those calculated by Springer et al. [2] using autocorrelated rates and soft bounds.

Hominina. We combined the matrix of 391 craniodental characters scored for late Miocene and Plio-Pleistocene homininans compiled by Dembo et al. [45] with the pedal character matrix (80 characters) compiled by DeSilva et al. [46] and ran a BTD analysis of the combined 471-character matrix. In the first iteration of the analysis, all pedal OTUs of DeSilva et al. that

matched a craniodental OTU in the Dembo et al. matrix were combined, and a BTD analysis was run. For all clock analyses we used the nonclock tree and the midpoints of the OTU's age prior to calculate a lognormal clockratepr parameter value of (-0.858,1.082) (i.e., using the R script for calculation of clockratepr from Gunnell et al. [1]). We used flat beta priors for extinctionpr and speciationpr parameters, and an exp(10) prior for speciationpr. *Pan* was constrained to be the sister taxon of *Homo* to the exclusion of *Gorilla*. The analysis was run for 35 million generations, with the first 25% discarded as burn-in. Based on the results of this analysis, if a pedal OTU in the matrix of DeSilva et al. that was not attributed to a known species formed a clade with a craniodental OTU, and were from the same time/place and of the appropriate size, the pedal OTU was combined with that craniodental OTU. A final BTD analysis was then run to obtain the final tree. For the final BTD analysis, we used the fixed tip dates derived from the original clock analysis and ran the analysis for 30 million generations, with the first 25% discarded as burn-in. The lowest effective sample size for any parameter was 293, and the ASDSF in the final generation was 0.004, providing strong evidence for convergence. Pedal OTUs that could not be justifiably attributed to a craniodental OTU were ultimately excluded from the tree that was grafted onto that derived from reanalysis of the matrix of Rasmussen et al. [29], as described above.

The primary topological differences from the results reported by Dembo et al. [45] are as follows: 1) placement of *Australopithecus africanus* closer to *Homo* than to *Paranthropus*, rather than as the sister taxon of *Paranthropus*; 2) placement of *Homo habilis* and *Homo floresiensis* as consecutive sister taxa of a clade containing *Homo rudolfensis*, *Homo erectus*, *Homo antecessor*, *Homo heidelbergensis*, *Homo neaderthalensis*, *Homo naledi*, and *Homo sapiens*, rather than being in a more basal position (*H. floresiensis*) or placed as the sister taxon of *Australopithecus sediba* (*Homo habilis*); and 3) placement of *Homo naledi* as the sister taxon of *Homo sapiens* to the exclusion of a *Homo heidelbergensis* + *Homo neaderthalensis* clade.

Note that as homininans fall far outside of the temporal window of interest for our study of dental topographic disparity, the inclusion of these homininan species serves only to increase the lineage diversity count through the Plio-Pleistocene in the composite tree, and has no impact on our results. The output of the final BTD analysis is provided in the Dryad repository associated with this study (https://doi.org/10.5061/dryad.pc866t1nw). Additional details and discussion of this analysis will be provided in another contribution that is currently in preparation.

Hystricognathi. Most fossil hystricognaths were placed into phylogenetic context using an augmented version of the matrix most recently used by Sallam and Seiffert [18]. To that matrix we added the early Oligocene species "*Phiomys stellae*", "*Phiomys troctatus*", *Acritophiomys* "*adaios*", and *Acritophiomys* "*woodi*" see [47], *Mubhammys atlanticus* [48], *Gaudeamus lavocati* [49], *Neophiomys dawsonae* [50], and the *Metaphiomys* species *zallahensis* [50] and *schaubi* [51], as well as the early Miocene species *Elmerimys woodi* and *Myophiomys arambourgi* [52]. We used all of the same parameter settings as Sallam and Seiffert [18], and ran the MCMC analysis for 25 million generations, with four chains and a temperature of 0.02. The smallest ESS for any parameter was 490.8 and the ASDSF in the final generation was 0.01, providing strong evidence for convergence of the runs. The input and output files for the analysis are available in the Dryad repository associated with this study (https://doi.org/10.5061/dryad.pc866t1nw).

The primary topological differences from the results reported by Sallam and Seiffert [18] are as follows: 1) placement of several basal hystricognaths (*Protophiomys*, *Waslamys*, advanced

baluchimyines) and *Acritophiomys* as a sister clade of the caviomorph *Canaanimys* + *Gaudeamus*, rather than as stem phiomorphs; 2) placement of phiocricetomyines as stem phiomorphs rather than falling outside of a Caviomorpha + Phiomorpha clade; 3) placement of *Tufamys* as a close relative of *Monamys*, rather than as basal petromurid; and 4) placement of *Prepomonomys* as a sister taxon of *Simonimys* + *Phiomyoides*, rather than as a sister taxon of *Diamantomys*.

Our justifications for additional grafted branches are as follows:

- Placement of extant bathyergoid genera is based on the molecular dating analysis of Patterson & Upham [53]. In their analysis, all of the relationships within Bathyergoidea were supported by a posterior probability of 1.0 (i.e. a *Cryptomys-Fukomys* clade, a *Georychus-Bathyergus* clade, placement of *Heliophobius* as sister of these clades, and placement of *Heterocephalus* (the sole member of the family Heterocephalidae) as the sister taxon of Bathyergidae).
- With the crown bathyergid clade fixed at 17.9 Ma, the only possible placements for the early Miocene (18-20 Ma [54]) bathyergoids *Renefossor*, *Bathyergoides*, *Geofossor*, *Microfossor*, and *Proheliophobius* are either as stem bathyergids or as stem heterocephalids. The only recent phylogenetic hypothesis for the placement of these fossil species is that of Mein and Pickford [54], who proposed that *Microfossor* was related to *Heterocephalus*, *Geofossor* was related to a *Cryptomys-Georychus* clade, and that *Efeldomys* was related to *Bathyergus*. However, the relationships among extant bathyergoids proposed by Mein and Pickford [54] are inconsistent with the very strong molecular evidence for their relationships and divergence times. The only placement of one of these early Miocene fossil bathyergoids that could be consistent with the molecular evidence presented by Patterson & Upham [53] is a placement of *Microfossor* as a sister taxon of *Heterocephalus*, a hypothesis which we have incorporated into our comprehensive tree. We placed *Renefossor* and *Bathyergoides* as sister taxa, given that *Renefossor* was erected for material that was previously treated as *Bathyergoides* [54]. *Efeldomys*, *Geofossor*, and *Proheliophobius* were placed arbitrarily as consecutive sister taxa of crown Bathyergidae. The tips for *Bathyergoides*, *Efeldomys*, *Geofossor*, and *Microfossor* were placed at 19 Ma, the midpoint of Mein & Pickford's 18-20 Ma estimate for the localities that yielded their remains, while *Proheliophobius* was assigned the mean of all the tip dates assigned Rusinga taxa (17.6 Ma) in our various BTD analyses. Lavocat [55] suggested that *Richardus* from Fort Ternan might be related specifically to *Heterocephalus*, a suggestion that we follow here.
- We used the relationships of extant bathyergid species recovered by Visser et al. [56]. The divergence dates among extant bathyergid genera presented by Visser et al. [56] are considerably older than those presented by Patterson and Upham [53], the latter of which are remarkably consistent with the fossil record of Phiomorpha [18]. As such, we have adjusted internal nodes within each bathyergid genus based on the proportional differences between the dates of Visser et al. [56] and Patterson and Upham [53] for each major split; specifically, branch lengths within *Heliophobius* were adjusted by 0.511, *Georychus* and *Bathyergus* by 0.49, and *Fukomys* by 0.388.
- The thryonomyoid *Pomonomys dubius* from Grillental, Namibia, is a specialized relative of *Diamantomys*. We place this species as the sister taxon of early Miocene *Diamantomys luederitzi*, and assign it a tip age of 19 Ma.

- Antonanzas et al. [57], found early middle Miocene *Paraphiomys orangeus* to be the sister taxon of *Paraulacodus* + *Thryonomys*. We place *P. orangeus* as the sister taxon of *Paraulacodus* + *Protohummus* + *Thryonomys*, with a tip age of 15 Ma.

Anomaluroidea, including Zegdoumyidae. In order to integrate zegdoumyids and anomaluroids, we ran a BTD analysis of the matrix of Marivaux et al. [58]. As in the other BTD analyses, we employed the fossilized birth-death prior, flat beta priors for the extinctionpr and fossilizationpr parameters, an exp(10) prior for speciationpr, and used the R script of Gunnell et al. [1] to calculate a value for clockratepr from the nonclock tree and the midpoints of each taxon's age prior [yielding a clockratepr prior of lognormal(-2.377,0.520)]. Because Marivaux et al.'s matrix includes stem rodents, we set the sampleprob value as 0.003, reflecting the fact that only 7 of the 2261 [59] extant rodent species were sampled in the matrix. The MCMC analysis was run for 25 million generations with 4 chains, and the first 25% of these generations were discarded as burn-in. The smallest effective sample size was 172 and the ASDSF was 0.005, providing strong evidence for convergence. The input and output files for the analysis are available in the Dryad repository associated with this study (https://doi.org/10.5061/dryad.pc866t1nw).

The primary topological differences from the parsimony-based results reported by Marivaux et al [58] are as follows: 1) placement of nementchamyids (including *Argouburus*, but excluding *Kabirmys prius*) as a clade of stem anomalurids, rather than as consecutive sister taxa of crown Anomaluroidea; 2) placement of *Nonanomalurus* + *Kabirmys prius* as stem anomalurids; and 3) placement of *Shazurus* and all species of *Paranomalurus* outside of crown Anomaluridae (as consecutive sister taxa), rather than being nested deep within that clade.

Hyaenodonta. Like rodents and primates, Hyaenodonta dispersed to Afro-Arabia during the early Paleogene. The oldest evidence of the clade on the continent is *Lahimia selloumi* from the middle to late Paleocene of the Ouled Abdoun Basin, Morocco [60]. The youngest occurrence of the clade is *Dissopsalis pyroclasticus* from the middle Miocene of Kenya [61]. The phylogenetic position of Hyaenodonta within Eutheria is traditionally resolved as being part of the clade Ferae [62]. Recent work on hyaenodont in-group relationships has consistently resolved a largely endemic Afro-Arabian clade of hyaenodonts called Hyainailouroidea, which includes nearly all Afro-Arabian taxa [63]. Digital dental models were not generated for the hyaenodont lineage because casts and scans of many key taxa are not yet available. Instead, the group was used to track lineage diversity through time for comparison with Afro-Arabian primates and rodents.

The BTD phylogenetic analysis is based on a modified matrix from Borths and Stevens [64] that includes updated character scoring from, and the addition of, a hyaenodont from Locality 41 in the Jebel Qatrani Formation of Egypt that is closely related to *Lahimia* and *Boualitomus*. The matrix has 100 total OTUs with 98 extinct hyaenodonts and 156 cranial, dental, and postcranial characters. As the entire lineage is extinct, molecular characters were not included. The tree that was used for LTT summation contains the 37 Afro-Arabian hyaenodont tips with the youngest tip in the analysis (*Dissopsalis pyroclasticus*) offset to ~11.7 Ma. The fossilized birth-death prior was implemented and the sampleprob prior was set at approximately zero. For all clock analyses we used the allcompat tree from a nonclock Bayesian analysis based on the results of the R script of Gunnell et al. [1] and the midpoints of the OTU's age prior to calculate a lognormal clockratepr parameter value of (-3.069,0.308) with a root age of 73 Ma, the midpoint for the oldest tip, *Maelestes*. The MCMC analysis was run 8 times for 500 million generations with 16 chains at a temperature of 0.05 sampling every 1000 generations, with the first 10% discarded for each run

as burn-in. The ASDSF was 0.0026, suggesting convergence on similar topological distributions across independent runs with ESS values ranging from 8600 to 43824. The tree, matrix, and all settings files are included in the Dryad repository associated with the study (https://doi.org/10.5061/dryad.pc866t1nw).

Supplementary References

1 Gunnell, G.F. *et al.* Fossil lemurs from Egypt and Kenya suggest an African origin for Madagascar's aye-aye. *Nature Communications* **9**, 3193 (2018).

2 Springer, M.S. *et al.* Macroevolutionary dynamics and historical biogeography of primate diversification inferred from a species supermatrix. *PLoS ONE* **7**, e49521 (2012).

3 Godinot, M. in *Anthropoid Origins* (eds Fleagle J.G. & Kay R.F.) 235-296 (Plenum Press, 1994).

4 Beard, K.C. & Wang, J. The eosimiid primates (Anthropoidea) of the Heti Formation, Yuanqu Basin, Shanxi and Henan Provinces, People's Republic of China. *Journal of Human Evolution* **46**, 401-432 (2004).

5 Hooker, J.J., Russell, D.E. & Phelizon, A. A new family of Plesiadapiformes (Mammalia) from the Old World lower Paleogene. *Palaeontology* **42**, 377-407 (1999).

6 Simons, E. L. & Bown, T. M. *Afrotarsius chatrathi,* first tarsiiform primate (?Tarsiidae) from Africa. *Nature* **313**, 475-477 (1985).

7 Jaeger, J.-J. *et al.* Late middle Eocene epoch of Libya yields earliest known radiation of African anthropoids. *Nature* **467**, 1096-1103 (2010).

8 Sallam, H.M. & Seiffert, E.R. New phiomorph rodents from the latest Eocene of Egypt, and the impact of Bayesian "clock"-based phylogenetic methods on estimates of basal hystricognath relationships and biochronology. *PeerJ* **4**, e1717 (2016).

9 Gheerbrant, E., Thomas, H., Roger, J., Sen, S. & Al-Sulaimani, Z. Deux nouveaux primates dans l'Oligocene inferieur de Taqah (Sultanat d'Oman): premiers adapiformes (?Anchomomyini) de la peninsule arabique? *Palaeovertebrata* **22**, 141-196 (1993).

10 Godinot, M., Senut, B. & Pickford, M. Primitive Adapidae from Namibia sheds light on the early primate radiation in Africa. *Communications of the Geological Survey of Namibia* **18**, 140-162 (2018).

11 Seiffert, E., Simons, E. & Attia, Y. Fossil evidence for an ancient divergence of lorises and galagos. *Nature* **422**, 421-424 (2003).

12 Seiffert, E. Early evolution and biogeography of lorisiform strepsirrhines. *American Journal of Primatology* **69**, 27-35 (2007).

13 Harrison, T. in *Cenozoic Mammals of Africa* (eds Lars Werdelin & William J. Sanders) 333-349 (University of California Press, 2010).

14 Pickford, M. *et al.* Refinement of the age of the middle Miocene Fort Ternan Beds, western Kenya, and its implications for Old World biochronology. *Comptes Rendus Geoscience* **338**, 545-555 (2006).

15 Kunimatsu, Y. *et al.* A new species of *Mioeuoticus* (Lorisiformes, Primates) from the early Middle Miocene of Kenya. *Anthropological Science* **125**, 59-65 (2017).

16 McCrossin, M. L. New species of bushbaby from the middle Miocene of Maboko Island, Kenya. *American Journal of Physical Anthropology* **89**, 215-233 (1992).

17 Marivaux, L. *et al.* A morphological intermediate between eosimiiform and simiiform primates from the late Middle Eocene of Tunisia: Macroevolutionary and paleobiogeographic implications of early anthropoids. *American Journal of Physical Anthropology* **154**, 387-401 (2014).

18 Sallam, H. M. & Seiffert, E. R. Revision of Oligocene '*Paraphiomys*' and an origin for crown Thryonomyoidea (Rodentia: Hystricognathi: Phiomorpha) near the Oligocene-Miocene boundary in Africa. *Zoological Journal of the Linnean Society* **190**, 352-371 (2020).

19 Marivaux, L. *et al.* A new and primitive species of *Protophiomys* (Rodentia, Hystricognathi) from the late middle Eocene of Djebel el Kébar, Central Tunisia. *Palaeovertebrata* **38**, 1-17 (2014).

20 de Bonis, L., Jaeger, J.-J., Coiffait, B. & Coiffait, P.-É. Découverte du plus ancien primate catarrhinien connu dans l'Eocène supérieur d'Afrique du Nord. *Comptes Rendus de l'Academie des Sciences Paris, Series II* **306**, 929-934 (1988).

21 Simons, E. L. & Kay, R. F. New material of *Qatrania* from Egypt with comments on the phylogenetic position of the Parapithecidae (Primates, Anthropoidea). *American Journal of Primatology* **15**, 337-347 (1988).

22 Ducrocq, S., Manthi, F.K. & Lihoreau, F. First record of a parapithecid primate from the Oligocene of Kenya. *Journal of Human Evolution* **61**, 327-331 (2011).

23 Beard, K.C., Coster, P.M.C., Salem, M.J., Chaimanee, Y. & Jaeger, J.-J. A new species of *Apidium* (Anthropoidea, Parapithecidae) from the Sirt Basin, central Libya: First record of Oligocene primates from Libya. *Journal of Human Evolution* **90**, 29-37 (2016).

24 Seiffert, E.R. *et al.* A parapithecid stem anthropoid of African origin in the Paleogene of South America. *Science* **368**, 194-197 (2020).

25 Seiffert, E.R. & Simons, E.L. Last of the oligopithecids? A dwarf species from the youngest primate-bearing level of the Jebel Qatrani Formation, northern Egypt. *Journal of Human Evolution* **64**, 211-215 (2013).

26 Simons, E. Egyptian Oligocene primates: A review. *Yearbook of Physical Anthropology* **38**, 199-238 (1995).

27 Thomas, H., Sen, S., Roger, J. & Al-Sulaimani, Z. The discovery of *Moeripithecus markgrafi* Schlosser (Propliopithecidae, Anthropoidea, Primates) in the Ashawq Formation (early Oligocene of Dhofar Province, Sultanate of Oman). *Journal of Human Evolution* **20**, 33-49 (1991).

28 Seiffert, E. R., Simons, E. L., Fleagle, J. G. & Godinot, M. in *Cenozoic Mammals of Africa* (eds Lars Werdelin & William J. Sanders) 369-392 (University of California Press, 2010).

29 Rasmussen, D.T. *et al.* Primitive Old World monkey from the earliest Miocene of Kenya and the evolution of cercopithecoid bilophodonty. *Proceedings of the National Academy of Sciences of the United States of America* **116**, 6051-6056 (2019).

30 Leakey, M. G., Ungar, P. S. & Walker, A. A new genus of large primate from the late Oligocene of Lothidok, Turkana District, Kenya. *Journal of Human Evolution* **28**, 519-531 (1995).

31 Stevens, N.J. *et al.* Palaeontological evidence for an Oligocene divergence between Old World monkeys and apes. *Nature* **497**, 611-614 (2013).

32 Zalmout, I.S. *et al.* New Oligocene primate from Saudi Arabia and the divergence of apes and Old World monkeys. *Nature* **466**, 360-364 (2010).

33 Miller, E.R. *et al.* Systematics of early and middle Miocene Old World monkeys. *Journal of Human Evolution* **57**, 195-211 (2009).

34 Rossie, J.B. & MacLatchy, L. A new pliopithecoid genus from the early Miocene of Uganda. *Journal of Human Evolution* **50**, 568-586 (2006).

35 Fleagle, J. G. & Seiffert, E. R. in *Evolution of Nervous Systems 2e* Vol. 3 (ed J. Kaas) 1–34 (Elsevier, 2017).

36 Pickford, M., Musalizi, S., Senut, B., Gommery, D. & Musiime, E. Small apes from the early Miocene of Napak, Uganda. *Geo-Pal Uganda* **3**, 1-111 (2010).

37 Cote, S., McNulty, K.P., Stevens, N.J. & Nengo, I.O. A detailed assessment of the maxillary morphology of *Limnopithecus evansi* with implications for the taxonomy of the genus. *Journal of Human Evolution* **94**, 83-91 (2016).

38 Rossie, J.B. & Hill, A. A new species of *Simiolus* from the middle Miocene of the Tugen Hills, Kenya. *Journal of Human Evolution* **125**, 50-58 (2018).

39 Nengo, I.O. *et al.* New infant cranium from the African Miocene sheds light on ape evolution. *Nature* **548**, 169-174 (2017).

40 Singleton, M. The phylogenetic affinities of *Otavipithecus namibiensis*. *Journal of Human Evolution* **38**, 537-573 (2000).

41 Ishida, H., Nakatsukasa, M., Kunimatsu, Y. & Nakano, Y. Erection of a new genus and species: *Nacholapithecus kerioi* for a middle Miocene hominoid from Nachola area, northern Kenya. *Anthropological Science* **108**, 92-92 (2000).

42 Suwa, G., Kono, R.T., Katoh, S., Asfaw, B. & Beyene, Y. A new species of great ape from the late Miocene epoch in Ethiopia. *Nature* **448**, 921-924 (2007).

43 Kunimatsu, Y. *et al.* A new Late Miocene great ape from Kenya and its implications for the origins of the African great apes and humans. *Proceedings of the National Academy of Sciences of the United States of America* **104**, 19220-19225 (2007).

44 Werdelin, L. in *Cenozoic Mammals of Africa* (eds Lars Werdelin & William J. Sanders) 27-43 (University of California Press, 2010).

45 Dembo, M. *et al.* The evolutionary relationships and age of *Homo naledi*: An assessment using dated Bayesian phylogenetic methods. *Journal of Human Evolution* **97**, 17-26 (2016).

46 DeSilva, J., McNutt, E., Benoit, J. & Zipfel, B. One small step: A review of Plio-Pleistocene hominin foot evolution. *American Journal of Physical Anthropology* **168**, 63-140 (2019).

47 Holroyd, P. A. *An examination of dispersal origins for Fayum Mammalia* Ph.D. thesis, Duke University, (1994).

48 Marivaux, L., Adnet, S., Benammi, M., Tabuce, R. & Yans, J. Earliest Oligocene hystricognathous rodents from the Atlantic margin of northwestern Saharan Africa (Dakhla, Morocco): Systematic, paleobiogeographical, and paleoenvironmental implications. *Journal of Vertebrate Paleontology* **37**, doi:10.1080/02724634.2017.1357567 (2017).

49 Coster, P. *et al. Gaudeamus lavocati* sp. nov. (Rodentia, Hystricognathi) from the early Oligocene of Zallah, Libya: first African caviomorph? *Naturwissenschaften* **97**, 697-706, doi:10.1007/s00114-010-0683-x (2010).

50 Coster, P. *et al.* A new early Oligocene mammal fauna from the Sirt Basin, central Libya: Biostratigraphic and paleobiogeographic implications. *Journal of African Earth Sciences* **104**, 43-55, doi:10.1016/j.jafrearsci.2015.01.006 (2015).

51 Wood, A. E. Early Cenozoic mammalian faunas, Fayum Province, Egypt, Part II: the African Oligocene Rodentia. *Peabody Museum Bulletin* **28**, 23-205 (1968).

52 Lavocat, R. Les Rongeurs du Miocène d'Afrique Orientale. *Mémoires et Travaux de l'Institut de Montpellier de l'École Pratique des Hautes Études*, 1-284 (1973).

53 Patterson, B. & Upham, N. A newly recognized family from the Horn of Africa, the Heterocephalidae (Rodentia: Ctenohystrica). *Zoological Journal of the Linnean Society* **172**, 942-963, doi:10.1111/zoj.12201 (2014).

54 Mein, P. & Pickford, M. Early Miocene Rodentia from the northern Sperrgebiet, Namibia. *Memoirs of the Geological Survey of Namibia* **20**, 235-290 (2008).

55 Lavocat, R. Osteologie de la tete de R*ichardus excavans* Lavocat 1988. *Palaeovertebrata* **19**, 73-80 (1989).

56 Visser, J., Bennett, N. & van Vuuren, B. Phylogeny and biogeography of the African Bathyergidae: a review of patterns and processes. *Peerj* **7**, doi:10.7717/peerj.7730 (2019).

57 Antonanzas, R., Sen, S. & Mein, P. Systematics and phylogeny of the cane rats (Rodentia : Thryonomyidae). *Zoological Journal of the Linnean Society* **142**, 423-444 (2004).

58 Marivaux, L., Adnet, S., Benammi, M. & Tabuce, R. Anomaluroid rodents from the earliest Oligocene of Dakhla, Morocco, reveal the long-lived and morphologically conservative pattern of the Anomaluridae and Nonanomaluridae during the Tertiary in Africa. *Journal of Systematic Palaeontology* **15**, 539-569, doi:10.1080/14772019.2016.1206977 (2017).

59 Fabre, P., Hautier, L., Dimitrov, D. & Douzery, E. A glimpse on the pattern of rodent diversification: a phylogenetic approach. *Bmc Evolutionary Biology* **12**, doi:10.1186/1471-2148-12-88 (2012).

60 Kocsis, L. *et al.* Comprehensive stable isotope investigation of marine biogenic apatite from the late Cretaceous-early Eocene phosphate series of Morocco. *Palaeogeography Palaeoclimatology Palaeoecology* **394**, 74-88, doi:10.1016/j.palaeo.2013.11.002 (2014).

61 Lewis, M. & Morlo, M. in *Cenozoic Mammals of Africa* (eds L. Werdelin & William J. Sanders) 543-560 (2010).

62 Halliday, T., Upchurch, P. & Goswami, A. Resolving the relationships of Paleocene placental mammals. *Biological Reviews* **92**, 521-550, doi:10.1111/brv.12242 (2017).

63 Borths, M., Holroyd, P. & Seiffert, E. Hyainailourine and teratodontine cranial material from the late Eocene of Egypt and the application of parsimony and

Bayesian methods to the phylogeny and biogeography of Hyaenodonta (Placentalia, Mammalia). *Peerj* **4**, doi:10.7717/peerj.2639 (2016).

64 Borths, M. & Stevens, N. Simbakubwa kutokaafrika, gen. et sp. nov. (Hyainailourinae, Hyaenodonta, 'Creodonta,' Mammalia), a gigantic carnivore from the earliest Miocene of Kenya. *Journal of Vertebrate Paleontology* **39**, doi:10.1080/02724634.2019.1570222 (2019).

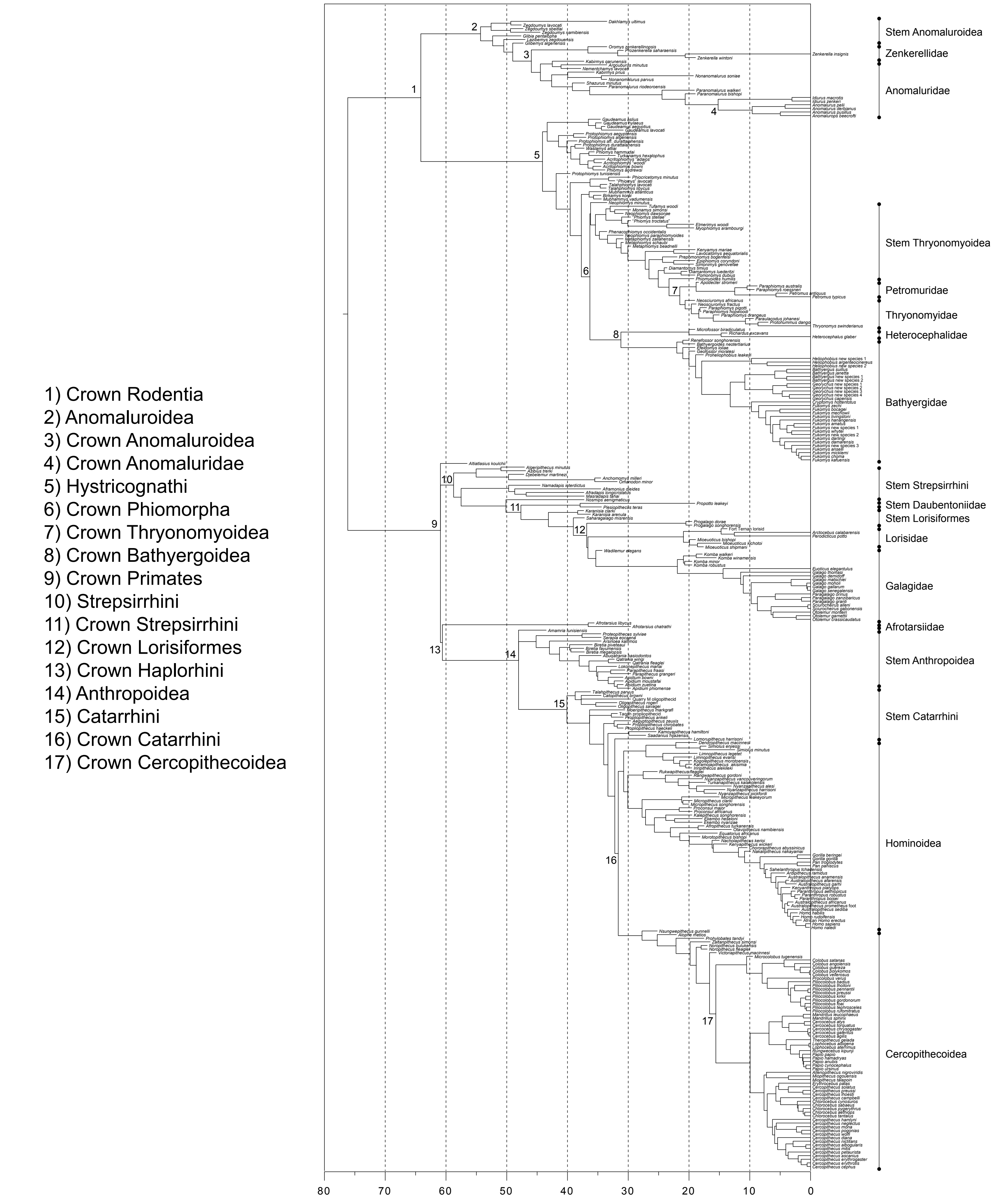

Supplementary Figure 1. Near-comprehensive composite phylogeny of African primates and rodents used for LTT plot in main text Figure 1B and 1C.

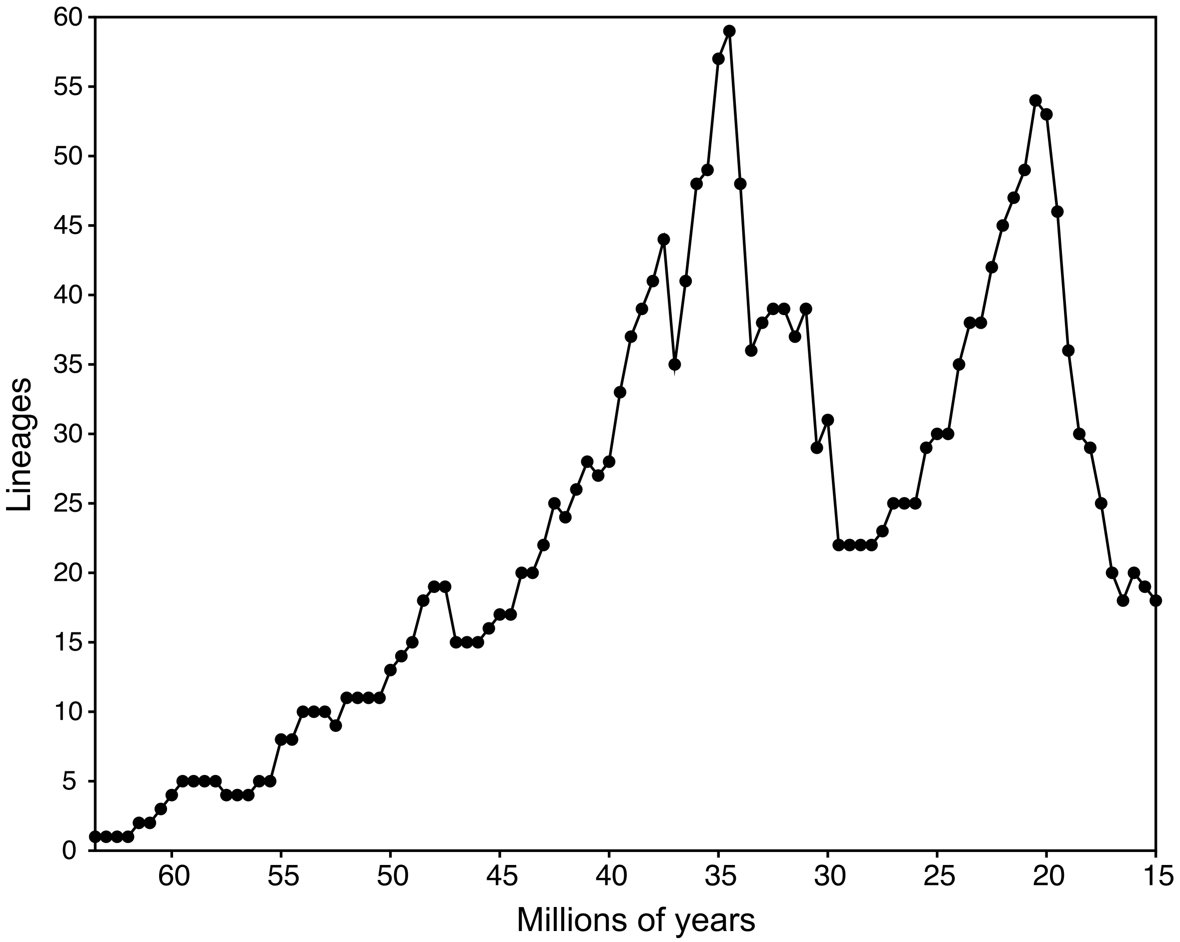

Supplementary Figure 2. Lineages-through-time plot for all five clades analyzed in this study (Anomaluroidea, Anthropoidea, Hystricognathi, Strepsirrhini, and Hyaenodonta) considered together.

ANOMALUROIDEA + HYSTRICOGNATHI

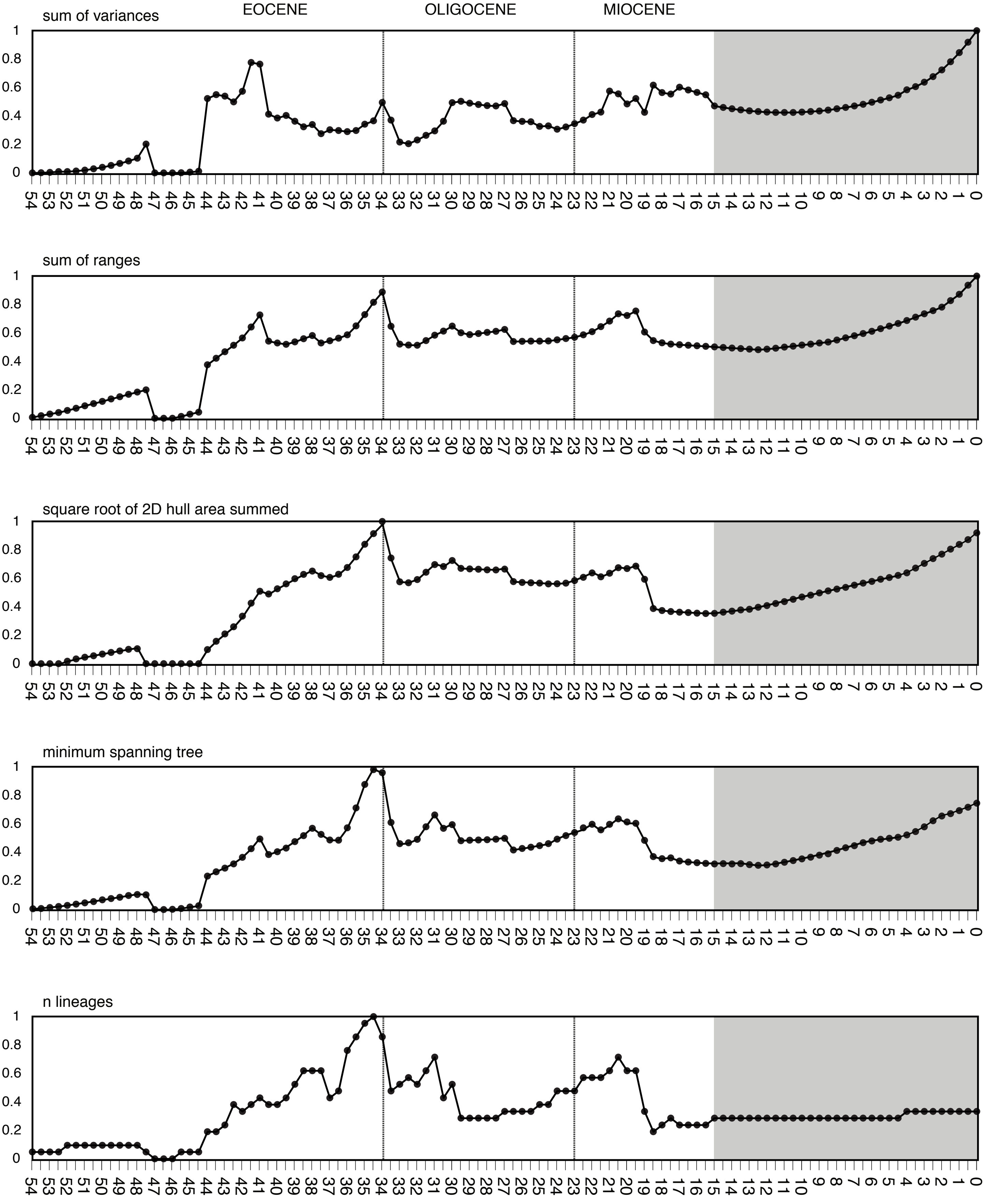

Supplementary Figure 3. Temporal changes in dental topographic disparity through time in endemic Afro-Arabian rodents (Anomaluroidea and Hystricognathi) from the Eocene to the present (top four plots), compared to temporal changes in lineage diversity (bottom plot). Disparity measures are based on principal components 1 and 2 of a principal components analysis of three dental topographic variables (ariaDNE, OPCR, and RFI). The grey box in each plot delimits an interval (post-15 Ma) for which no fossil taxa were sampled.

ANOMALUROIDEA

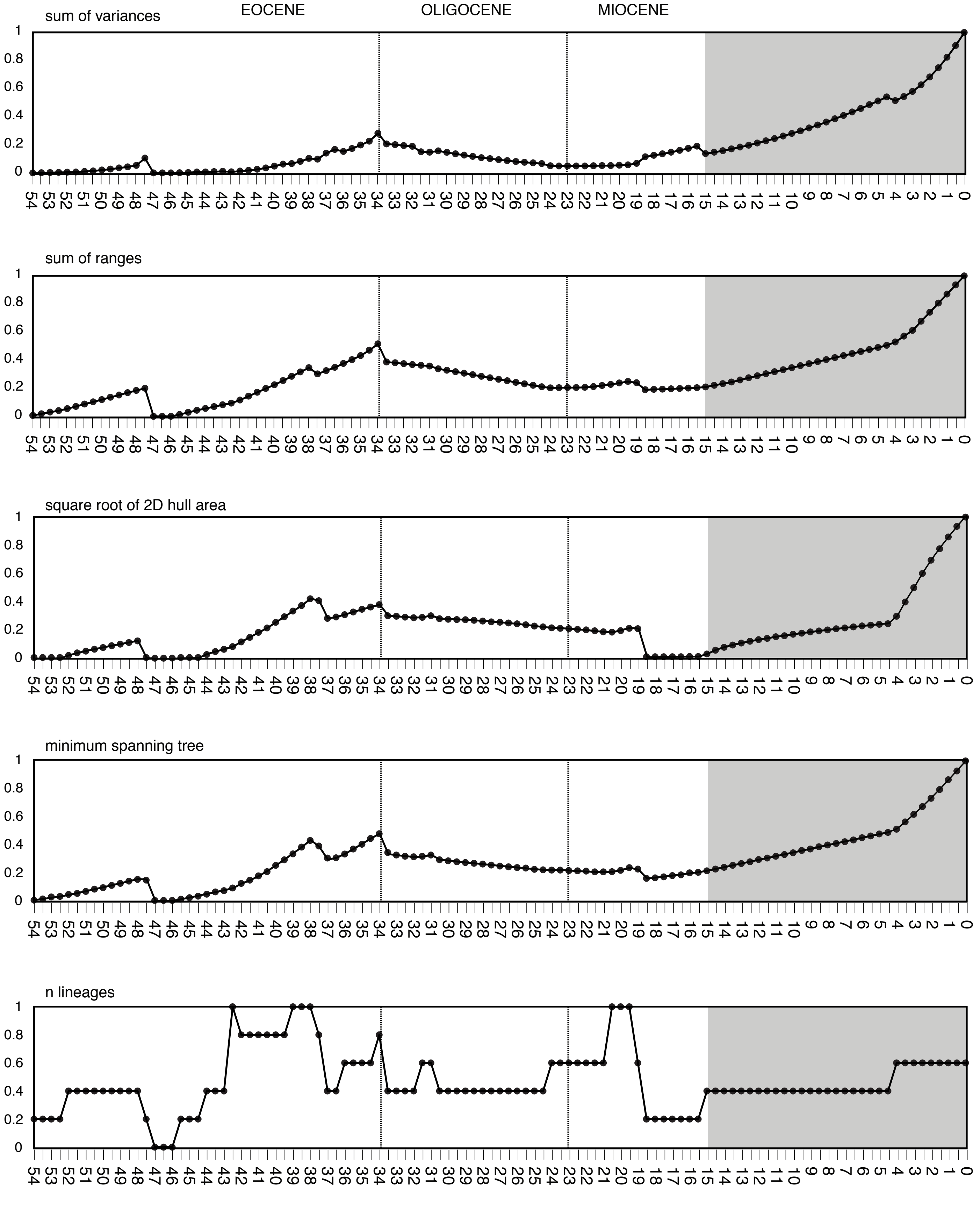

Supplementary Figure 4. Temporal changes in dental topographic disparity through time in Afro-Arabian anomaluroid rodents from the Eocene to the present (top four plots), compared to temporal changes in lineage diversity (bottom plot). Disparity measures are based on principal components 1 and 2 of a principal components analysis of three dental topographic variables (ariaDNE, OPCR, and RFI). The grey box in each plot delimits an interval (post-15 Ma) for which no fossil taxa were sampled.

HYSTRICOGNATHI

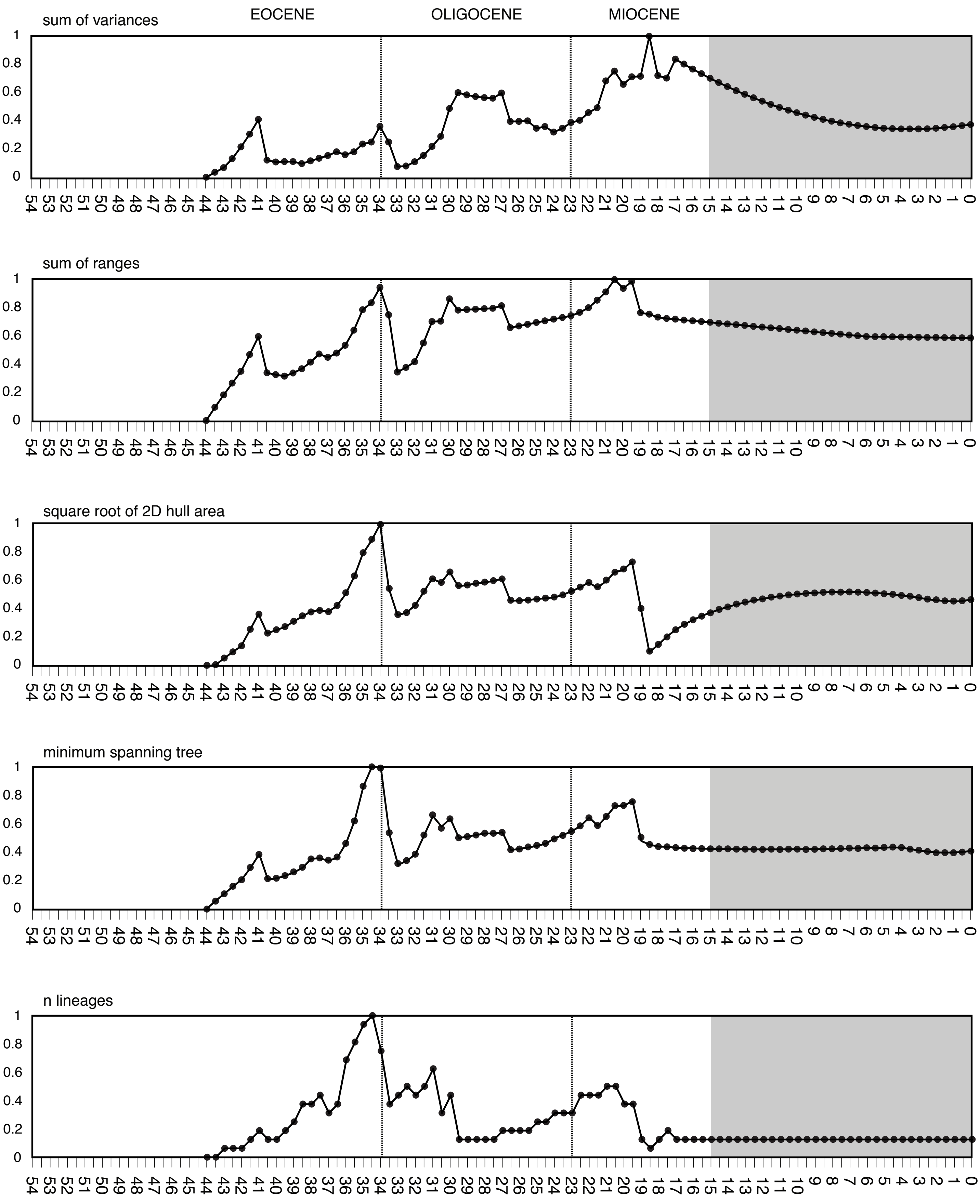

Supplementary Figure 5. Temporal changes in dental topographic disparity through time in Afro-Arabian hystricognath rodents from the Eocene to the present (top four plots), compared to temporal changes in lineage diversity (bottom plot). Disparity measures are based on principal components 1 and 2 of a principal components analysis of three dental topographic variables (ariaDNE, OPCR, and RFI). The grey box in each plot delimits an interval (post-15 Ma) for which no fossil taxa were sampled.

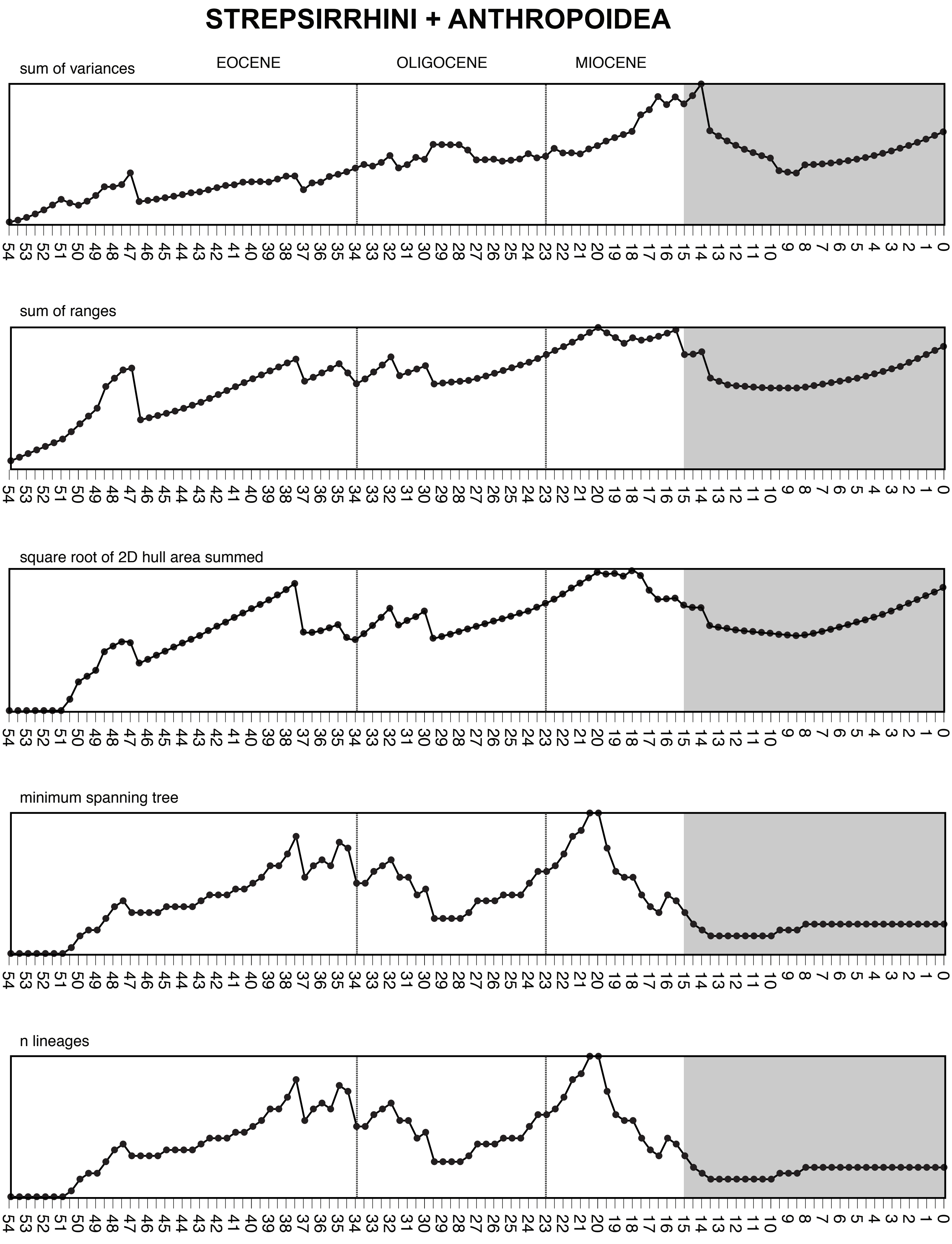

Supplementary Figure 6. Temporal changes in dental topographic disparity through time in endemic Afro-Arabian primates (Strepsirrhini and Anthropoidea) from the Eocene to the present (top four plots), compared to temporal changes in lineage diversity (bottom plot). Disparity measures are based on principal components 1 and 2 of a principal components analysis of three dental topographic variables (ariaDNE, OPCR, and RFI). The grey box in each plot delimits an interval (post-15 Ma) for which no fossil taxa were sampled.

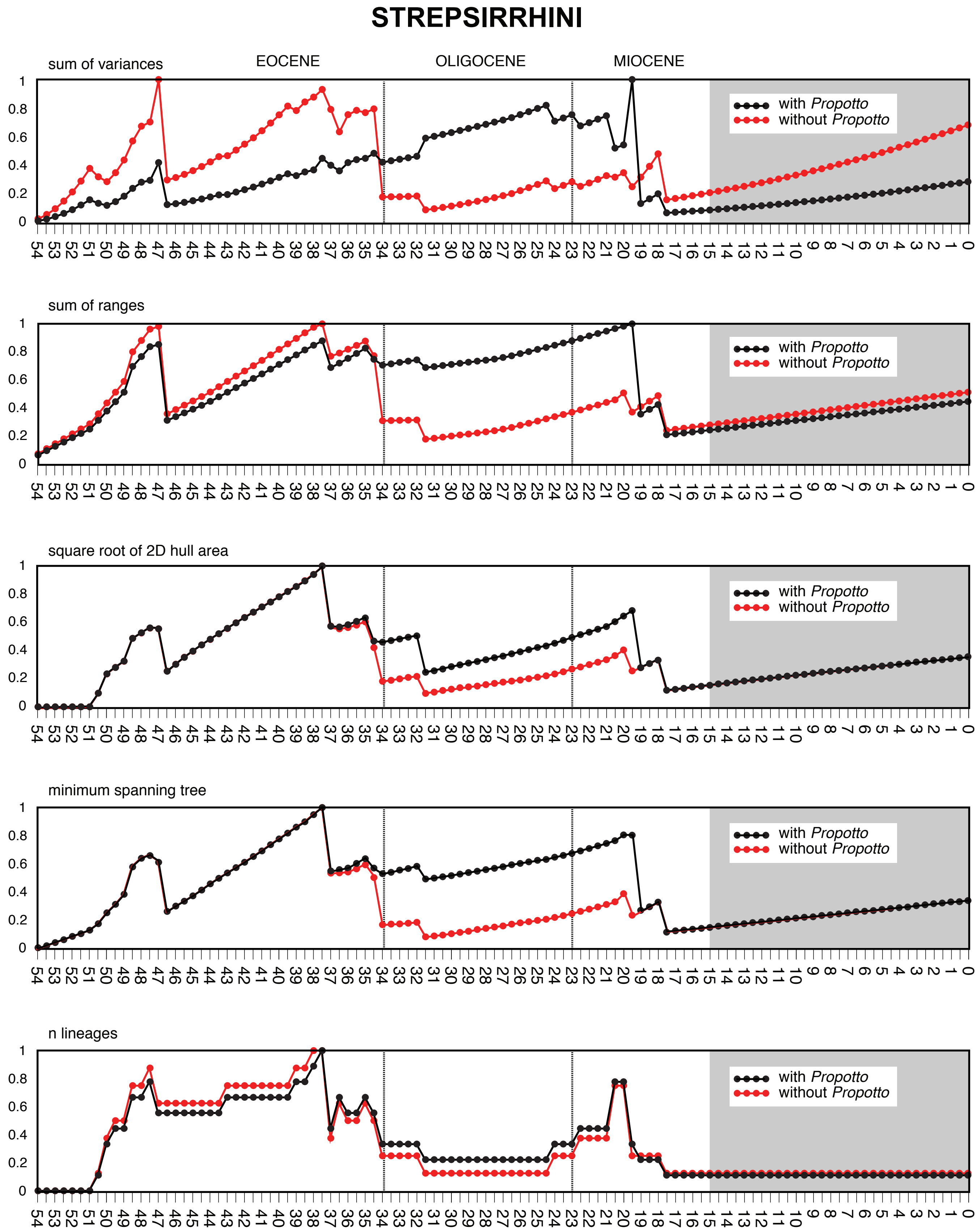

Supplementary Figure 7. Temporal changes in dental topographic disparity through time in Afro-Arabian strepsirrhines from the Eocene to the present (top four plots), compared to temporal changes in lineage diversity (bottom plot). Disparity measures are based on principal components 1 and 2 of a principal components analysis of three dental topographic variables (ariaDNE, OPCR, and RFI). The grey box in each plot delimits an interval (post-15 Ma) for which no fossil taxa were sampled. For each plot temporal trends are shown with (black lines and circles) and without (red lines and circles) the morphologically divergent early Miocene strepsirrhine *Propotto*.

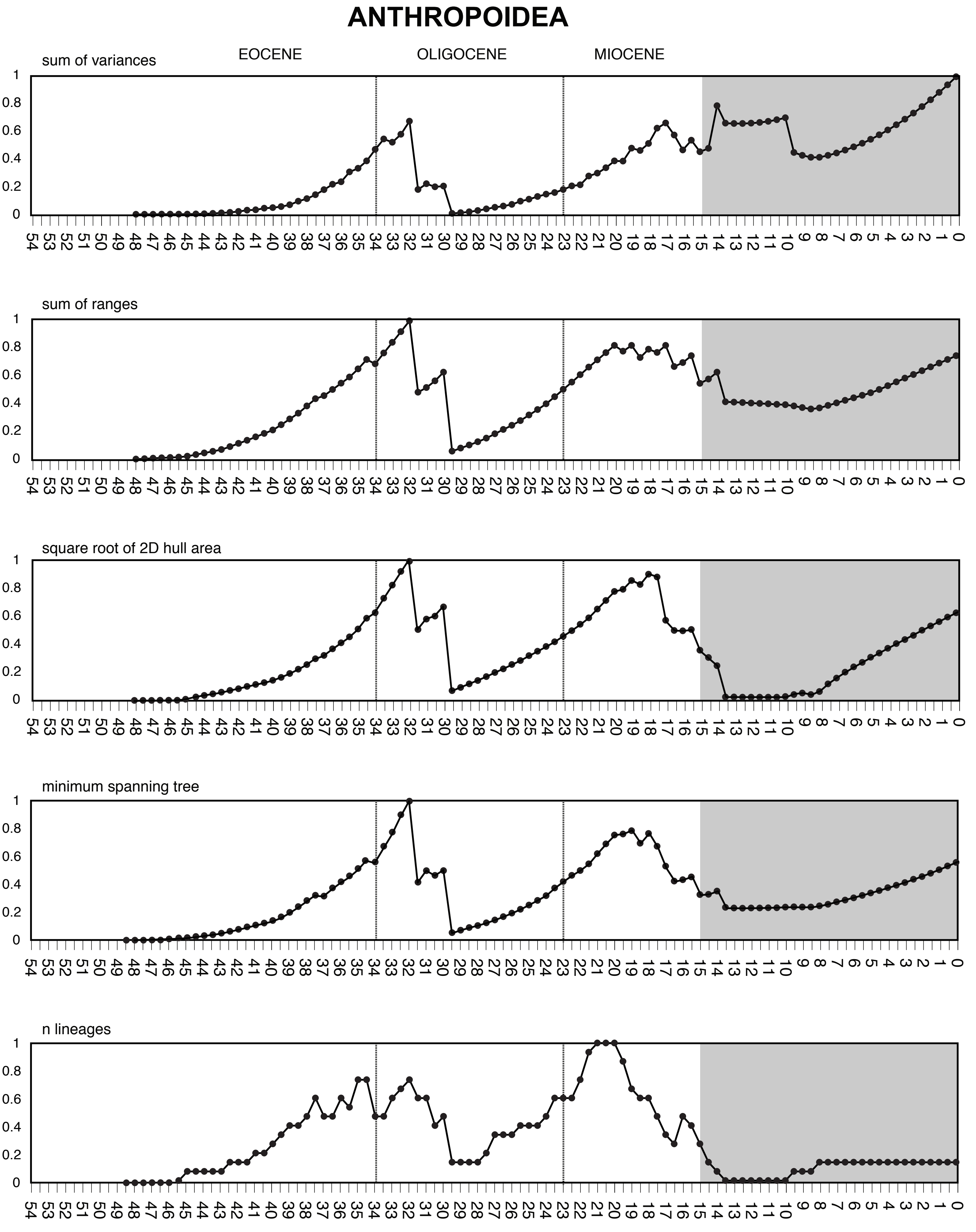

Supplementary Figure 8. Temporal changes in dental topographic disparity through time in Afro-Arabian anthropoids from the Eocene to the present (top four plots), compared to temporal changes in lineage diversity (bottom plot). Disparity measures are based on principal components 1 and 2 of a principal components analysis of three dental topographic variables (ariaDNE, OPCR, and RFI). The grey box in each plot delimits an interval (post-15 Ma) for which no fossil taxa were sampled.
