# Supplementary material for: Widespread loss of mammalian lineage and dietary diversity in the early Oligocene of Afro-Arabia
Source: Commun Biol. 2021 Oct 7;4:1172. doi: 10.1038/s42003-021-02707-9 (PMC8497553; doi:10.1038/s42003-021-02707-9)
Supplement: Supplementary file 2 — Reporting summary. [file 42003_2021_2707_MOESM2_ESM.pdf]

Corresponding author(s): Erik R. Seiffert
Last updated by author(s): Sep 8, 2021

Reporting Summary

Nature Portfolio wishes to improve the reproducibility of the work that we publish. This form provides structure for consistency and transparency in reporting. For further information on Nature Portfolio policies, see our Editorial Policies and the Editorial Policy Checklist.

Statistics

For all statistical analyses, confirm that the following items are present in the figure legend, table legend, main text, or Methods section.

n/a	Confirmed	
☐	☒	The exact sample size (*n*) for each experimental group/condition, given as a discrete number and unit of measurement
☒	☐	A statement on whether measurements were taken from distinct samples or whether the same sample was measured repeatedly
☒	☐	The statistical test(s) used AND whether they are one- or two-sided<br>*Only common tests should be described solely by name; describe more complex techniques in the Methods section.*
☒	☐	A description of all covariates tested
☒	☐	A description of any assumptions or corrections, such as tests of normality and adjustment for multiple comparisons
☒	☐	A full description of the statistical parameters including central tendency (e.g. means) or other basic estimates (e.g. regression coefficient) AND variation (e.g. standard deviation) or associated estimates of uncertainty (e.g. confidence intervals)
☒	☐	For null hypothesis testing, the test statistic (e.g. *F*, *t*, *r*) with confidence intervals, effect sizes, degrees of freedom and *P* value noted<br>*Give P values as exact values whenever suitable.*
☐	☒	For Bayesian analysis, information on the choice of priors and Markov chain Monte Carlo settings
☒	☐	For hierarchical and complex designs, identification of the appropriate level for tests and full reporting of outcomes
☒	☐	Estimates of effect sizes (e.g. Cohen's *d*, Pearson's *r*), indicating how they were calculated

Our web collection on statistics for biologists contains articles on many of the points above.

Software and code

Policy information about availability of computer code

Data collection	Phylogenetic results were output from MrBayes 3.2.7. Dental topographic data were collected in MorphoTester. AriaDNE was calculated using MATLAB scripts available at https://github.com/sshanshans/ariaDNE_code. Dental topographic models were prepared in Avizo and GeoMagic. Phylogenetic constraints were created using the R package paleotree. LTT plots were created using the R package ape. Principal components analyses were performed in R using the 'prcomp()' function from the 'stats' package. Ancestral state reconstructions were performed in mvMORPH. Calculation of SR2DHA used the function concaveman() from the R package concaveman. Polygon areas were calculated using the function Polygon from the R package sp. MST point associations were found using the function mst() from the R package ape.
Data analysis	All R code was developed specifically for this project. Analyses were run in R 4.0.2. All code has been made available in the Dryad repository associated with the study (https://doi.org/10.5061/dryad.pc866t1nw).

For manuscripts utilizing custom algorithms or software that are central to the research but not yet described in published literature, software must be made available to editors and reviewers. We strongly encourage code deposition in a community repository (e.g. GitHub). See the Nature Portfolio guidelines for submitting code & software for further information.

Data

Policy information about availability of data

All manuscripts must include a data availability statement. This statement should provide the following information, where applicable:

- Accession codes, unique identifiers, or web links for publicly available datasets
- A description of any restrictions on data availability
- For clinical datasets or third party data, please ensure that the statement adheres to our policy

Input data files, settings, code and results from phylogenetic, ASR, and disparity analyses are available on the Dryad Digital Repository (https://doi.org/10.5061/

dryad.pc866t1nw). Digital surface models for all of the figured fossil specimens are available on MorphoSource (www.morphosource.org), except those housed at the National Museums of Kenya that are currently under review before uploading.

Field-specific reporting

Please select the one below that is the best fit for your research. If you are not sure, read the appropriate sections before making your selection.

☐ Life sciences ☐ Behavioural & social sciences ☒ Ecological, evolutionary & environmental sciences

For a reference copy of the document with all sections, see nature.com/documents/nr-reporting-summary-flat.pdf

Ecological, evolutionary & environmental sciences study design

All studies must disclose on these points even when the disclosure is negative.

Study description	Our study includes 1) construction of a composite phylogeny of Afro-Arabian anthropoid and strepsirrhine primates and anomaluroid and hystricognath rodents using a combination of Bayesian tip-dating techniques and grafting of unsampled species; 2) calculation of 10K randomized trees using the same tip ages; 3) Bayesian tip-dating analysis of Hyaenodonta, based on a morphological character matrix; 4) calculation of dental topographic metrics (OPCR, RFI, ariaDNE) on 329 second lower molar specimens of 134 species; 5) principal components analysis of the dental topographic metrics; 6) calculation of ancestral state reconstructions of the dental topographic metrics on the composite phylogeny; 7) time-slice trait interpolation on the composite tree; 8) time-slice characterization of disparity through time.
Research sample	The composite phylogeny includes 317 tips. Of these, 134 species were sampled for dental topographic metrics.
Sampling strategy	We sampled as many species as was possible given budgetary and time constraints, and the willingness of other researchers to share casts or scans.
Data collection	DdV collected data used for the dental topographic analyses. Whenever possible, microCT scans of original specimens were used. Whenever this was not possible due to limited time and budget, casts were made of original specimens. High resolution molds were made using President Jet Plus Light Body impression material (Polyvinylsiloxane-based, manufactured by Coltene Whaledent) and casts were made using Epo-Tek 301 (epoxy, manufactured by Epoxy Technology). Molds and casts were made following protocols that have yielded high resolution copies of dentition that were used in micro-wear studies that require features at the micrometre scale to be copied (e.g. Ungar et al. 2006, JHE: 50).<br>microCT scans were used to create digital surface files of the dentition in Avizo v8.0. Three-dimensional surface files were processed following extensively tested protocols outlined by Spradley et al. 2017 (AJPA: 163) and Berthaume et al. 2019 (PloS one, 14).<br>Digital surface files were used to calculate the dental topographic metrics. MorphoTester was used to calculate OPCR, surface area, and surface outline area. Surface area and surface outline were used to calculate RFI in excel. ariaDNE (epsilon = 0.1) was calculated in MatLab using code made publicly available by Shan et al. (2019, Methods in Ecology and Evolution: 10).
Timing and spatial scale	Data were collected and processed from May 2017 up to July 2020.
Data exclusions	*If no data were excluded from the analyses, state so OR if data were excluded, describe the exclusions and the rationale behind them, indicating whether exclusion criteria were pre-established.*
Reproducibility	Reproducibility and the inter-personal error of manually orienting the tooth into occlusal view was tested by De Vries (2020, PhD Thesis, Table 2.1 and 2.2) and was found to be 2.4% for OPCR, 0.5% for RFI, and 0.09% for DNE; all of these errors were well below the within-species variation observed in dental topographic values.
Randomization	*Describe how samples/organisms/participants were allocated into groups. If allocation was not random, describe how covariates were controlled. If this is not relevant to your study, explain why.*
Blinding	*Describe the extent of blinding used during data acquisition and analysis. If blinding was not possible, describe why OR explain why blinding was not relevant to your study.*

Did the study involve field work? ☐ Yes ☒ No

Reporting for specific materials, systems and methods

We require information from authors about some types of materials, experimental systems and methods used in many studies. Here, indicate whether each material, system or method listed is relevant to your study. If you are not sure if a list item applies to your research, read the appropriate section before selecting a response.

Materials & experimental systems

n/a	Involved in the study
☒	☐ Antibodies
☒	☐ Eukaryotic cell lines
☐	☒ Palaeontology and archaeology
☒	☐ Animals and other organisms
☒	☐ Human research participants
☒	☐ Clinical data
☒	☐ Dual use research of concern

Methods

n/a	Involved in the study
☒	☐ ChIP-seq
☒	☐ Flow cytometry
☒	☐ MRI-based neuroimaging

Palaeontology and Archaeology

Specimen provenance: No fieldwork was carried out as part of this study. All fossil specimens included in this study are vouchered, are located in museums and/or research institutions, and are available for study.

Specimen deposition: All fossil specimens included in this study are vouchered, are located in museums and/or research institutions, and are available for study.

Dating methods: N/A

☐ Tick this box to confirm that the raw and calibrated dates are available in the paper or in Supplementary Information.

Ethics oversight: No ethical approval or guidance was needed as all fossils were considered geological samples.

Note that full information on the approval of the study protocol must also be provided in the manuscript.
